# Free-breathing simultaneous native myocardial T1, T2 and T1ρ mapping with Cartesian acquisition and dictionary matching

**DOI:** 10.1186/s12968-023-00973-6

**Published:** 2023-11-09

**Authors:** Zhenfeng Lyu, Sha Hua, Jian Xu, Yiwen Shen, Rui Guo, Peng Hu, Haikun Qi

**Affiliations:** 1 School of Biomedical Engineering, ShanghaiTech University, 4th Floor, BME Building, 393 Middle Huaxia Road, Pudong District, Shanghai, 201210 China; 2 Shanghai Clinical Research and Trial Center, Shanghai, China; 3 Department of Cardiovascular Medicine, Ruijin Hospital Lu Wan Branch, Shanghai Jiao Tong University School of Medicine, Shanghai, China; 4 UIH America, Inc., Houston, TX USA; 5 School of Medical Technology, Beijing Institute of Technology, Beijing, China

**Keywords:** Multi-parametric mapping, Free breathing, Dictionary matching, T1ρ mapping

## Abstract

**Background:**

T1, T2 and T1ρ are well-recognized parameters for quantitative cardiac MRI. Simultaneous estimation of these parameters allows for comprehensive myocardial tissue characterization, such as myocardial fibrosis and edema. However, conventional techniques either quantify the parameters individually with separate breath-hold acquisitions, which may result in unregistered parameter maps, or estimate multiple parameters in a prolonged breath-hold acquisition, which may be intolerable to patients. We propose a free-breathing multi-parametric mapping (FB-MultiMap) technique that provides co-registered myocardial T1, T2 and T1ρ maps in a single efficient acquisition.

**Methods:**

The proposed FB-MultiMap performs electrocardiogram-triggered single-shot Cartesian acquisition over 16 consecutive cardiac cycles, where inversion, T2 and T1ρ preparations are introduced for varying contrasts. A diaphragmatic navigator was used for prospective through-plane motion correction and the in-plane motion was corrected retrospectively with a group-wise image registration method. Quantitative mapping was conducted through dictionary matching of the motion corrected images, where the subject-specific dictionary was created using Bloch simulations for a range of T1, T2 and T1ρ values, as well as B1 factors to account for B1 inhomogeneities. The FB-MultiMap was optimized and validated in numerical simulations, phantom experiments, and in vivo imaging of 15 healthy subjects and six patients with suspected cardiac diseases.

**Results:**

The phantom T1, T2 and T1ρ values estimated with FB-MultiMap agreed well with reference measurements with no dependency on heart rate. In healthy subjects, FB-MultiMap T1 was higher than MOLLI T1 mapping (1218 ± 50 ms vs. 1166 ± 38 ms, p < 0.001). The myocardial T2 and T1ρ estimated with FB-MultiMap were lower compared to the mapping with T2- or T1ρ-prepared 2D balanced steady-state free precession (T2: 41.2 ± 2.8 ms vs. 42.5 ± 3.1 ms, p = 0.06; T1ρ: 45.3 ± 4.4 ms vs. 50.2 ± 4.0, p < 0.001). The pathological changes in myocardial parameters measured with FB-MultiMap were consistent with conventional techniques in all patients.

**Conclusion:**

The proposed free-breathing multi-parametric mapping technique provides co-registered myocardial T1, T2 and T1ρ maps in 16 heartbeats, achieving similar mapping quality to conventional breath-hold mapping methods.

**Supplementary Information:**

The online version contains supplementary material available at 10.1186/s12968-023-00973-6.

## Background

Quantitative cardiac magnetic resonance (MR) parametric mapping has emerged as a promising non-invasive tool for characterizing myocardial tissue, diagnosing various cardiovascular diseases, and monitoring treatment effects [1–3]. The relaxation times or parameters, such as commonly used T1 and T2, represent intrinsic tissue characteristics in a magnetic field and can reflect pathological changes of tissue. Inflammation, fibrosis and amyloid deposition lead to increased native T1 values [4–6], while the opposite can be observed in conditions such as iron deposition or substantial fat accumulation [7–9]. Elevated T2 values reflect increased free water content in the tissue, typically caused by myocardial edema or inflammation [10]. T1ρ, on the other hand, represents the longitudinal relaxation in a rotating frame, under a spin lock (SL) radiofrequency pulse which has a low frequency of several hundred Hertz. T1ρ has been shown to provide endogenous contrast for changes in macromolecular content [11]. Previous studies found elevated T1ρ in acute and chronic myocardial infarction [12–17], as well as in nonischemic myocardial diseases, such as dilated cardiomyopathy [18] and hypertrophic cardiomyopathy [19]. Compared with native T1, T1ρ may be a more promising endogenous contrast for myocardial fibrosis, as increased T1 is not specific to fibrosis and the ability of native T1 to detect myocardial fibrosis is still controversial.

T1, T2 and T1ρ parameters provide complementary information about the myocardium, and a combination of multiple parameters may improve diagnostic sensitivity and boost diagnostic confidence for suspected cardiomyopathy [3, 20]. However, myocardial parameters are commonly measured individually in separate breath-hold acquisitions [1, 21–25], which may result in nonregistered maps, prolonged scan time, and cause patient discomfort due to repeated breath-holds throughout the sequential acquisitions. In addition, the separate mapping methods such as MOdified Look Locker Inversion recovery (MOLLI) for T1 mapping [21] or T2-prepared balanced steady-state free precession (bSSFP) for T2 mapping [24] require dummy cardiac cycles to allow for longitudinal magnetization recovery needed for better exponential curve fitting, which results in inefficient acquisition and heart rate dependency.

Simultaneous multi-parametric mapping techniques have been proposed to overcome the limitations of separate mapping methods. Previous cardiac multi-parametric mapping approaches are mostly for T1 and T2 mapping, which can be generally classified into two categories. The first category involves sophisticated sequence design by interleaving saturation, inversion and T2 preparation pulses so that the acquired multi-contrast images can be fitted to an analytical formula for T1 and T2 mapping [26–31]. The second category of techniques, such as cardiac MR fingerprinting (MRF) [32], involves designing a sequence that generates different signal evolutions for different T1 and T2 combinations [33–38]. Subsequently, the acquired signals are matched to a dictionary generated according to the imaging sequence in order to derive the quantitative parameters. The dictionary matching techniques are not limited to acquiring images that conform to certain analytical function and thus can be easily extended to mapping more parameters. Velascol et al. have extended the cardiac MRF technique for simultaneous T1, T2 and T1ρ mapping [39]. However, besides the long breath-hold of 16 heartbeats, the MRF technique adopts highly undersampled spiral trajectory to sample the signal evolution and requires complex iterative reconstruction before dictionary matching to remove aliasing artifacts. Recently, the feasibility of single-shot Cartesian dictionary-based mapping has been demonstrated for myocardial T1 and T2 mapping, where only 10 time-points along the magnetization evolution were sampled. Cartesian dictionary-based mapping involves simplified post-processing and is more ready for clinical translation. However, the current Cartesian dictionary-based T1 and T2 mapping technique still requires breath-hold to mitigate respiratory motion and assumes homogeneous B1 across the left ventricle. Adding more parameters to be quantified would lead to increased scan time that may exceed the limit of breath-hold duration. Furthermore, there may be B1 inhomogeneities, especially at high field scanners. If not accounted for, the inhomogeneous B1 will influence the accuracy of dictionary-based parameter estimations.

The objective of this study is to propose a free-breathing multi-parametric mapping (FB-MultiMap) technique that enables B1 inhomogeneity-corrected simultaneous myocardial T1, T2 and T1ρ mapping with single-shot Cartesian acquisition and dictionary matching. Inspired by previous studies that leverage the diaphragmatic navigator (dNAV) for free-breathing parameter mapping [31, 40, 41], the dNAV was adopted in FB-MultiMap for through-plane motion correction with special design to reduce the influence of preparation pulses on the dNAV signal. The residual in-plane motion was corrected using a multi-contrast group-wise registration method [42]. The effects of spatial inhomogeneities of the radiofrequency transmit field, slice profile and respiratory motion-induced deviations between the measured signal and the simulated signal evolutions were modelled as an effective B1 factor in the dictionary of FB-MultiMap to achieve B1-corrected multi-parametric mapping. The proposed technique was optimized and validated in numerical simulations, phantoms, healthy subjects and patients with suspected cardiomyopathy.

## Methods

**Fig. 1:**
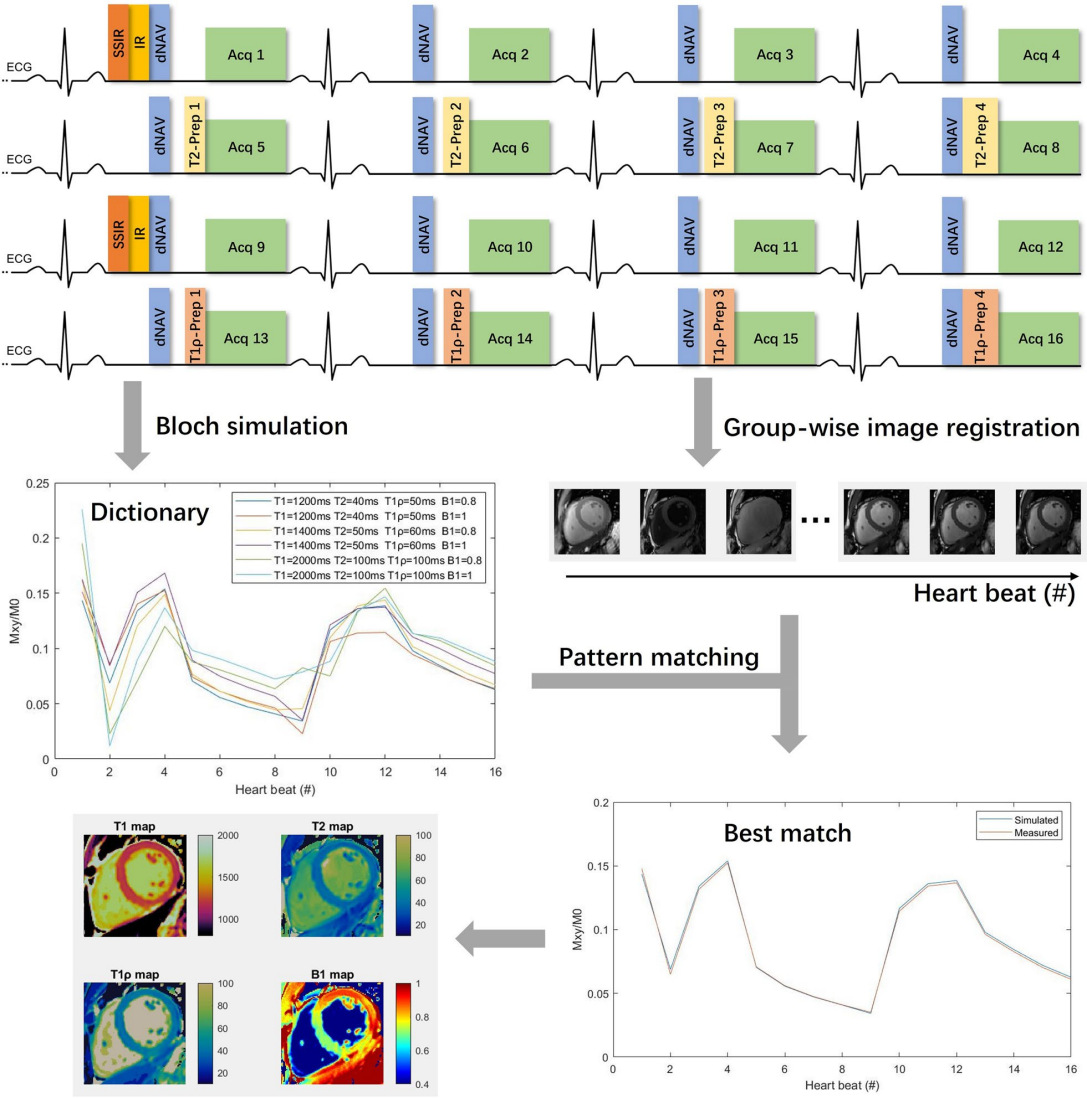
Sequence diagram and post-processing steps for the proposed free-breathing multi-parametric mapping technique with Cartesian sampling and dictionary matching. Sixteen cardiac cycles are consecutively acquired using inversion recovery (IR), T2 preparation (T2-prep) and T1ρ preparation (T1ρ-prep) pulses to introduce T1, T2 and T1ρ sensitization, respectively. The diaphragm navigator (dNAV) is performed for prospective respiratory motion correction by updating the imaging plane in real-time. The slice-selective IR (SSIR) is applied to restore the diaphragm signal. After image acquisition, the in-plane motion is retrospectively corrected using group-wise image registration. Dictionary matching is subsequently performed to find the dictionary entry that best matches the acquired signal at each pixel for T1, T2, T1ρ and B1 mapping

An overview of the proposed free-breathing multi-parametric mapping technique is provided in Fig. 1, which includes single-shot acquisition with the optimized FB-MultiMap sequence, multi-contrast image registration, dictionary generation with Bloch simulations and T1, T2, T1ρ and B1 mapping with dictionary matching. Details are provided in the following sections.

### Sequence optimization

The proposed free-breathing multi-parametric mapping sequence performs electrocardiogram-triggered single-shot Cartesian acquisition with 2D bSSFP readout including five ramp-up pulses to reach steady-state and linear k-space ordering, where inversion recovery (IR), T2 preparation (T2-prep) and T1ρ preparation (T1ρ-prep) pulses are included for T1, T2 and T1ρ sensitization, respectively. The T2-prep includes hard 90° tip-down and tip-up pulses and two adiabatic refocusing pulses [24]. The T1ρ-prep module adopts the 90°_x_-SL_y_-SL_-y_-180°_-x_-SL_-y_-SL_y_-90°_x_ pulses cluster, where the SL pulse is equally divided into four segments. As shown in the previous T1ρ-prep optimization studies [43, 44], this T1ρ-prep is robust to B1 and B0 field inhomogeneities and performs better than the totally balanced spin-lock module with two 180° refocusing pulses [14, 45].

The accuracy and precision of quantitative measurements can be affected by the number and timings of preparation pulses and an exhaustive search for the optimal design may be intractable. Several candidate sequences with different preparation pulse configurations were empirically designed for investigation. Details of sequence optimization are provided in the Additional file 1.

FB-MultiMap adopts the bSSFP readout with large flip angles, and the inhomogeneous transmit B1 field, if not accounted for, would influence the dictionary-based mapping accuracy [36, 46]. However, with the Look-Locker type acquisition, the effect of excitation pulses with constant flip angle and T1 relaxation on the inversion recovered signal cannot be reliably separated [47]. Therefore, we proposed to change the flip angle after IR [48] and estimate the B1 factor along with T1, T2 and T1ρ to correct the nominal flip angle. To determine appropriate flip angle combinations, numerical simulations, phantom and in vivo experiments were conducted. In simulations, the flip angle was varied from 35° to 70° with a step of 5°. The target T1, T2, T1ρ and B1 were set to 1200 ms, 40 ms, 50 ms and 0.8, respectively. The relative root mean squared error (RRMSE) for T1, T2 and T1ρ, and the mean RRMSE of the three parameters were computed to evaluate the performance of FB-MultiMap with different flip angle combinations in simulation and phantom studies. The calculation equation of RRMSE is provided in the Additional file 1. For in vivo imaging, the relative spatial variability (RSD) was calculated by dividing the standard deviation (SD) with the mean value in the myocardium for T1, T2 and T1ρ.

### Motion correction

For respiratory motion correction, the cross-pair diaphragm navigator was utilized to adjust the imaging plane in real-time to correct the through-plane motion with an empirical tracking factor of 0.6 [41, 49]. dNAV based respiratory motion correction has previously been adopted for free-breathing myocardial mapping [31, 40]. The preparation pulses in FB-MultiMap are all non-selective. To reduce the influence of IR on the dNAV signal, a slice-selective IR was performed covering the imaging location of dNAV along with the non-selective IR to restore the magnetization in the right diaphragm region. Also, the dNAV was applied before T2-prep and T1ρ-prep to avoid their influence. After image acquisition, a group-wise registration method proposed to correct motion for MOLLI T1 mapping [50] was adopted to correct in-plane motion among the multi-contrast images.

### Parametric mapping

Dictionary matching was performed for parametric mapping. The subject-specific dictionary was generated with recorded R-wave intervals and trigger delays using the Bloch equation for ranges of T1, T2, T1ρ and B1. To evaluate the effect of correcting B1, FB-MultiMap with simplified post-processing (sFB-MultiMap) without modelling B1 in the dictionary simulation was also performed. Different from MRF which simulates the magnetization for each readout, for the Cartesian sampling, only one image was acquired per cardiac cycle with the contrast dominated by the k-space center and therefore only the transverse magnetization simulated corresponding to the center of k-space was used for the dictionary. Considering that online reconstructed images are usually magnitude images without phase information, the magnitude of the simulated magnetization was recorded for the dictionary to avoid determining the polarity of the magnitude images. In our initial experiments, we found dictionary matching with and without polarity achieved similar accuracy and precision for the proposed multi-parametric mapping technique.

### Phantom experiments

The proposed FB-MultiMap sequence was implemented and evaluated on a 3T United Imaging scanner (uMR 890, United Imaging Healthcare, Shanghai, China) with 12-channel body and 48-channel spine coils. The phantoms with T1, T2 and T1ρ corresponding to physiological ranges were made of different concentrations of agarose and gadolinium-based contrast agent. Reference T1 values were obtained using IR spin echo with 11 inversion recovery times from 35 to 3000 ms and TR of 7000 ms. Reference T2 values were measured using multi-echo spin echo with 10 TEs ranging from 14 to 140 ms and TR of 7000 ms. Reference T1ρ values were estimated using a T1ρ-prep gradient echo technique [51] with spin lock time (TSL) = [2, 16, 30, 50, 80] ms and spin-lock frequency = 350 Hz. The reference images were acquired with 2 × 2 mm^2^ spatial resolution and 8 mm slice thickness. The multi-parametric mapping scans were performed with simulated heart rates from 20 to 120 bpm with 20 bpm increments, to test heart rate dependency. Other imaging parameters were: field-of-view (FOV) = 320 × 280 mm^2^; pixel size = 2.08 × 1.67 mm^2^; slice thickness = 8 mm; TR/TE = 2.82 ms/1.33 ms; readout bandwidth = 1200 Hz/pixel; inversion recovery time = 255 ms; generalized autocalibrating partially parallel acquisition (GRAPPA) [52] parallel imaging acceleration = 2, resulting in 77 lines acquired per heartbeat.

To reduce computation cost, the dictionary in the phantom studies was generated considering the actual parameters ranges. Specifically, the dictionary contained approximately 6,037,000 T1/T2/T1ρ/B1 combinations: [10:10:1800] ms for T1; [10:2:160] for T2 and T1ρ; [0.4:0.05:0.55, 0.55:0.02:0.8, 0.8:0.05:1.2] for B1. Combinations of T2 > T1 and T1ρ > T1 were excluded from the dictionary simulation. The Bloch simulations were implemented using MATLAB (The MathWorks, Natick, Massachusetts) and the dictionary generation took about 6 min on a workstation (Intel Xeon Gold 6226R 2.9 GHz processor, 384 Gb RAM) with parallel computing.

### In vivo experiments

The in vivo imaging was approved by the local institutional review board. Fifteen healthy subjects (6 females, age: 26 ± 3.3 years, heart rate: 71 ± 7.7 bpm) and 6 patients with suspected cardiomyopathy (1 females, age: 58 ± 19.9 years, heart rate: 72.5 ± 15.3 bpm) were imaged after obtaining written informed consent. The demographic information and referral reasons of the patients are summarized in Table 1.

**Table 1:** Demographic information of healthy subjects and patients

	n	Age (year)	Height (cm)	Weight (kg)	BMI
Volunteers	15 (F = 6)	26 ± 3.3	171 ± 6.7	69 ± 11	24 ± 2.4
Patients	6 (F = 1)	58 ± 19.9	170 ± 6.0	66 ± 13	23 ± 4.5

Patient referral reasons					
Fabry disease		3	Hypertensive cardiomyopathy		1
Old myocardial infarction		1	Cardiac amyloidosis		1

The optimized FB-MultiMap was evaluated against the breath-hold separate mapping methods: MOLLI 5(3)3 for T1 mapping [21], T2-prep bSSFP for T2 mapping [24] and T1ρ-prep bSSFP for T1ρ mapping [51]. T2-prep bSSFP acquired three T2-weighted images with T2-prep durations = [0, 35, 55] ms, while T1ρ-prep bSSFP acquired four T1ρ-weighted images with TSL = [2, 16, 30, 50] ms and spin-lock frequency of 350 Hz. Both T2-prep bSSFP and T1ρ-prep bSSFP employed three idle cardiac cycles between each readout for signal recovery. Furthermore, to assess any respiratory motion induced parameter estimation variability of FB-MultiMap, the optimized multi-parameter mapping sequence was also performed under breath-holding in the healthy subjects, who are able to hold their breath for a relatively long time. Three short-axis slices at basal, middle and apical left ventricle were acquired for all mapping techniques. Other imaging parameters, including the matrix size, slice thickness and readout bandwidth, were the same to those of FB-MultiMap in phantom studies. The FOV in the phase-encoding direction was adjusted according to the subject’s size in the range of 240–280 mm. In addition to the mapping scans, the late gadolinium enhancement (LGE) imaging was performed in the patients after the mapping acquisitions to detect any focal enhancement. The breath-hold T1ρ-prep bSSFP was not included in the patients imaging protocol to reduce the number of breath-holds.

The dictionary generated for in vivo studies contained about 2,330,000 T1/T2/T1ρ/B1 combinations: [500:100:800, 810:10:1700, 1710:100:2000] ms for T1, [10:10:30, 31:2:70, 71:5:91, 91:10:121] for T2 and T1ρ, and [0.4:0.05:0.5, 0.5:0.03:1.1, 1.1:0.05:1.2] for B1. It took about 1.5 min for the subject-specific dictionary generation and 10 s for extracting parametric maps per slice using dictionary matching.

### Image analysis

For phantoms, the mean and SD were calculated for each tube. The estimations of FB-MultiMap and sFB-MultiMap were then respectively compared with the reference methods using Pearson correlation. Bland–Altman analysis was also conducted to evaluate the agreement of FB-MultiMap with the reference methods. For in vivo images, region-of-interests were manually defined in the T1, T2 and T1ρ maps according to the 16-segment of the American Heart Association (AHA) model [53]. Subsequently, the mean and SD were calculated for each short-axis slice to evaluate the estimation accuracy and precision in healthy subjects. The slice-wise mean and SD were compared between the four measurement methods including separate breath-hold mapping, breath-hold simultaneous multi-parametric mapping (BH-MultiMap), FB-MultiMap and sFB-MultiMap using one-way ANOVA (One-way analysis of variance) with Bonferroni post-hoc correction. p < 0.05 is considered statistically significant.

## Results

### Sequence optimization

The optimized FB-MultiMap is demonstrated in Fig. 1, where the IR pulses are played at the first and the ninth cardiac cycles; T2-preps are played in the fifth to eighth cardiac cycles with durations = [35, 45, 55, 65] ms; T1ρ-prep with TSLs = [16, 30, 40, 50] ms are played in the 13th to 16th cardiac cycles. Detailed sequence optimization results are provided in the Additional file 1.

**Fig. 2:**
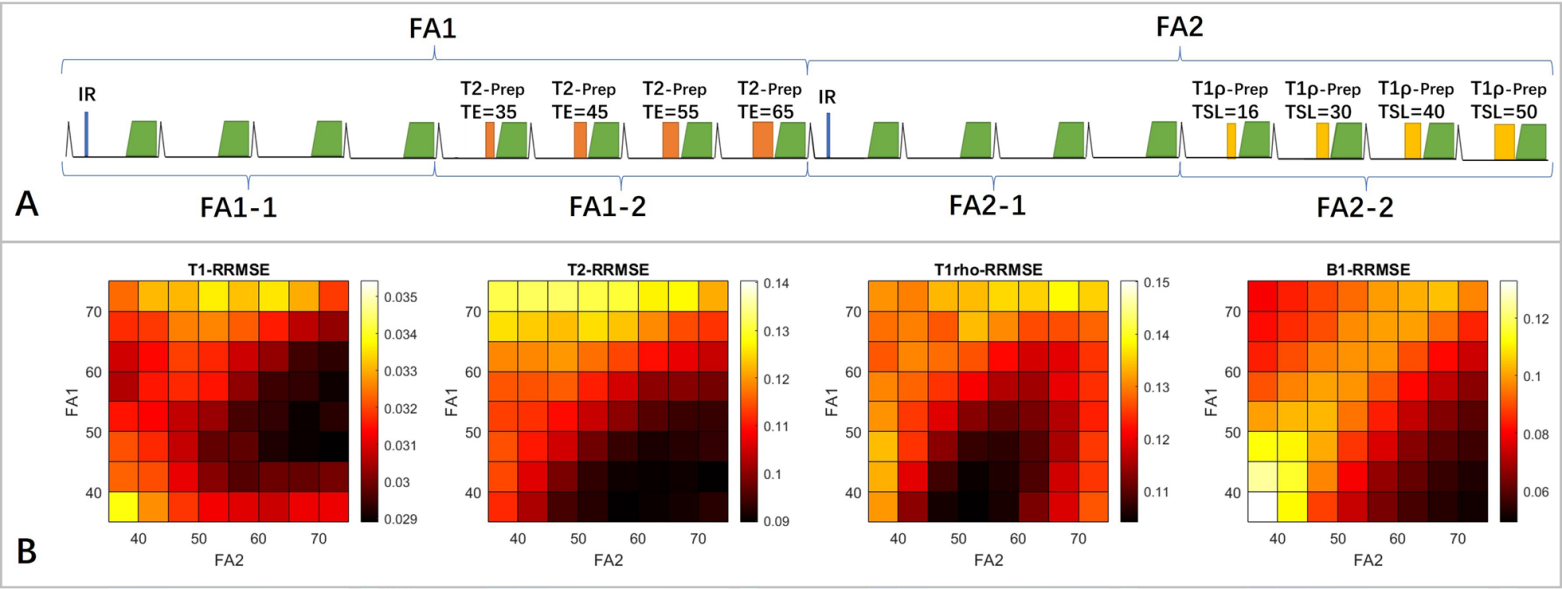
**A** illustrates the two different variable flip angle (VFA) strategies. The first strategy involves changing the flip angle after the second IR resulting in two flip angles (*FA1*, *FA2*). The second strategy involves changing the flip angle after the first T2-prep, second IR, and first T1ρ-prep leading to four flip angles (*FA1-1*, *FA1-2*, *FA2-1*, and *FA2-2*) in total. **B** shows the relative root mean-squared error (RRMSE) of numerical simulations for the dual flip angle scheme with different combinations of *FA1* and *FA2*

**Fig. 3:**
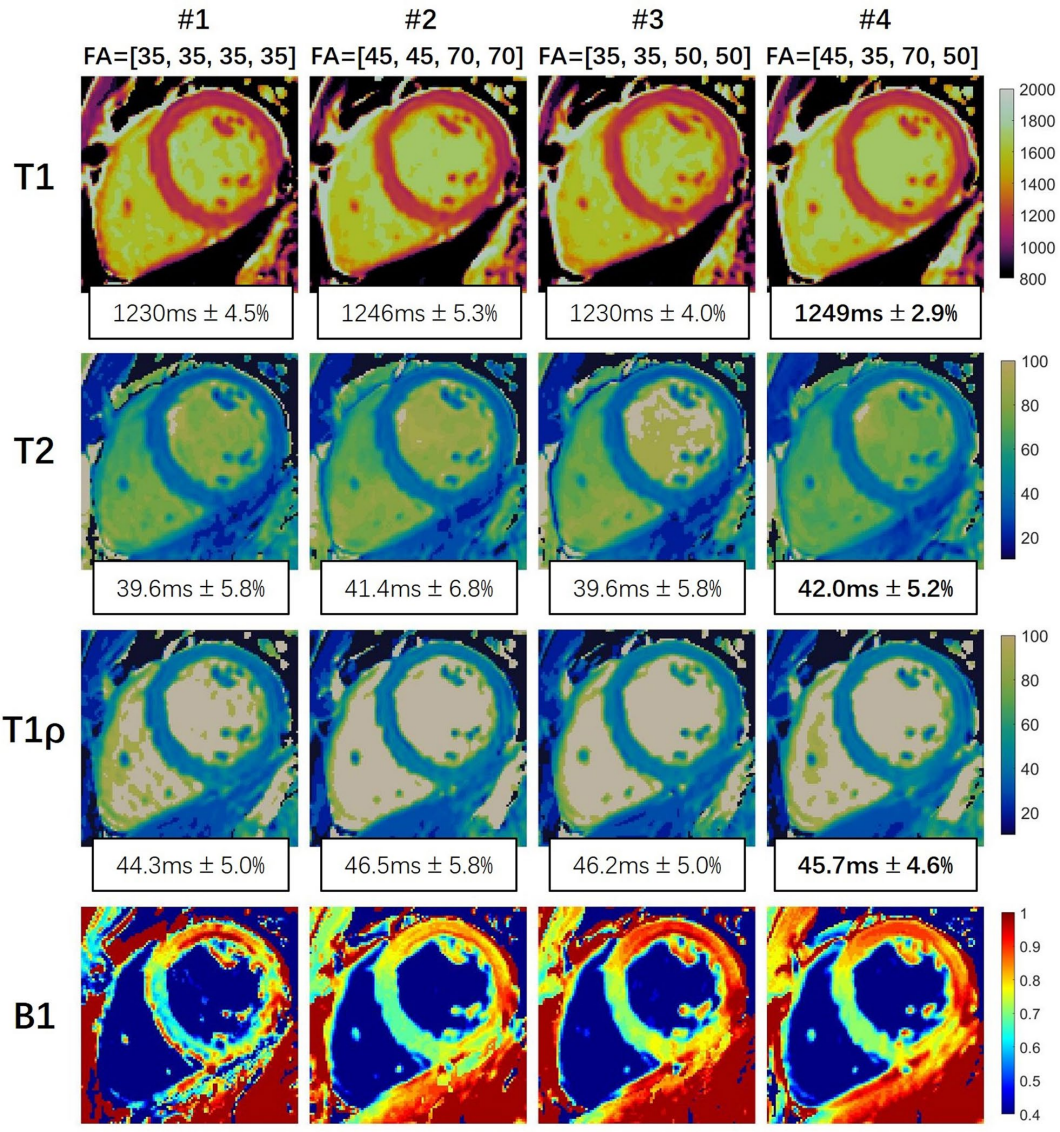
T1, T2, T1ρ and B1 maps generated using the proposed FB-MultiMap with different flip angle combinations in a healthy subject. The mean and relative standard deviation (RSD) in the myocardium were calculated and reported under each parameter map. The optimized four flip angle strategy achieved the lowest RSD for all parameters. It is noted that B1 in the blood pool cannot be reliably estimated due to blood flowing

For the variable flip angle strategy, the dual flip angle scheme was initially tested with the excitation flip angle changed after the second IR (*FA1*/*FA2* in Fig. 2A). However, as shown in the RRMSE maps in Fig. 2B, no *FA1*/*FA2* combinations resulted in the lowest RRMSE for all parameters, with T1 favoring 45°/70°, while T2 and T1ρ favoring low *FA1* of 35° and moderate *FA2* of 50°. Therefore, we subsequently proposed to also alter the flip angles after the first T2-prep and the first T1ρ-prep pulse, resulting in a four-flip-angle strategy, *FA1-1*, *FA1-2*, *FA2-1* and *FA2-2* in Fig. 2A. With the observation that T1 and B1 RRMSE were mostly influenced by *FA1-1* and *FA2-1*, while T2 and T1ρ RRMSE was respectively more influenced by *FA1-2* and *FA2-2*, the appropriate four flip angle combination can be inferred from the dual flip angle results with *FA1-1* (45°) and *FA2-1* (70°) being the optimal flip angles for T1 and B1 and *FA1-2* (35°) and *FA2-2* (50°) being the optimal flip angles for T2 and T1ρ. The inferred four-flip-angle strategy (45°/35°/70°/50°) avoids the brute-force search for the optimal combination of four flip angles that would require prohibitively long computation time. Figure 3 shows example T1, T2, T1ρ and B1 maps of a healthy subject estimated with FB-MultiMap with different flip angle combinations. The variable flip angle schemes resulted in smoother B1 maps than the constant flip angle scheme, and the optimized four flip angle scheme outperformed the dual flip angle scheme by achieving lower spatial variability (RSD) for all three parameters. It is noted that B1 in ventricular blood pool cannot be reliably estimated due to blood flowing. Additional results of simulations and phantoms regarding variable flip angle optimization are included in the Additional file 1.

### Phantom experiments

**Fig. 4:**
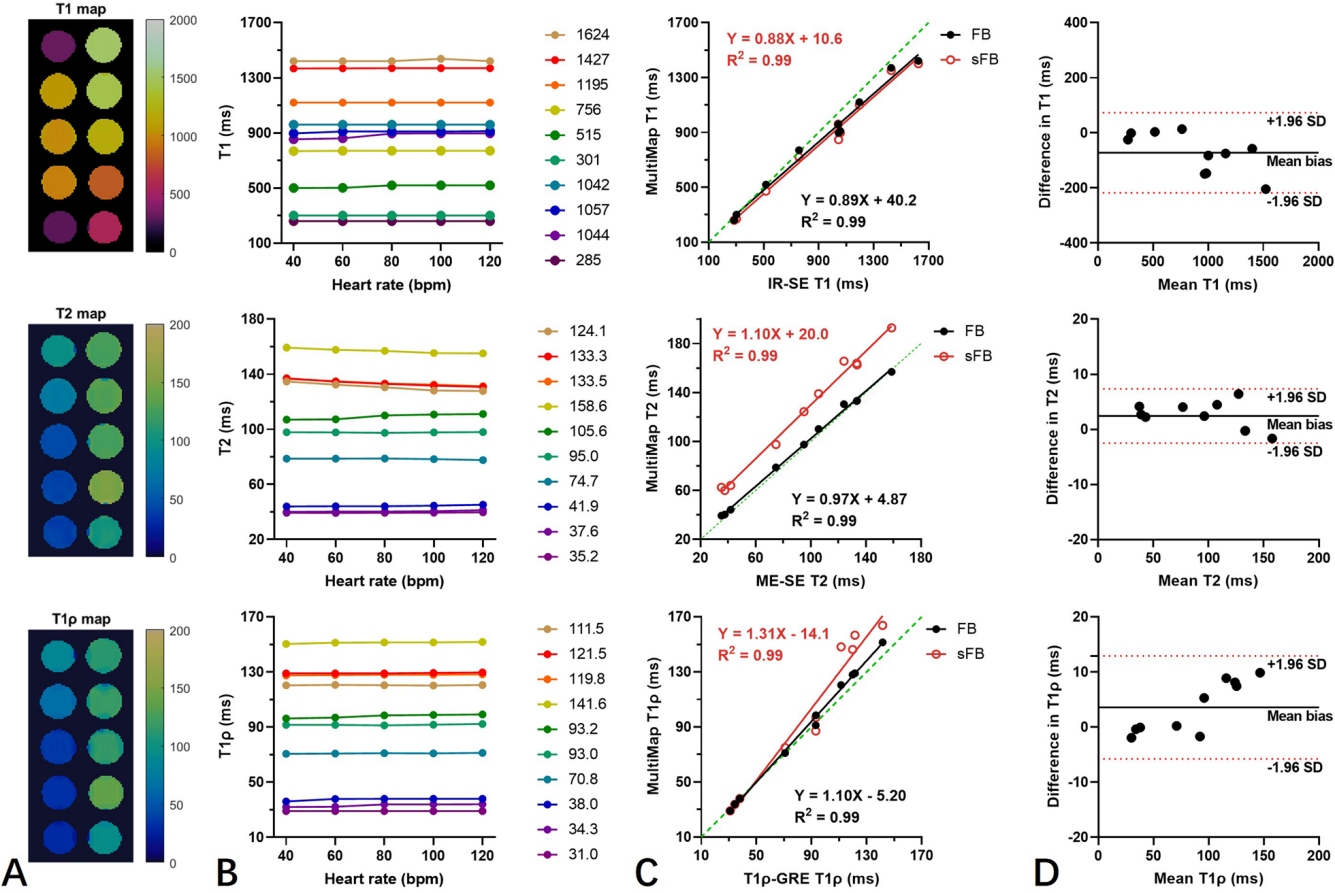
Phantom results. **A** Example phantom T1, T2 and T1ρ maps estimated by FB-MultiMap with simulated heart rate of 80 bpm. **B** T1, T2 and T1ρ estimated with FB-MultiMap at simulated heart rates from 40 to 120 bpm with the reference values showing in the legend. **C** Correlation of FB-MultiMap and sFB-MultiMap T1, T2 and T1ρ measured at heart rate of 80 bpm with the reference values. **D** The Bland–Altman analyses, showing the difference between FB-MultiMap and the reference method along with their average. The black solid lines indicate the measurement bias: T1 bias = − 71.9 ms; T2 bias = 1.8 ms; T1ρ bias = 3.9 ms

Example phantom T1, T2 and T1ρ maps estimated with FB-MultiMap at heart rate of 80 bpm are shown in Fig. 4A. FB-MultiMap estimations were overall consistent for heart rates ranging from 40 to 120 bpm (Fig. 4B) and showed strong correlation with the reference values (correlation coefficients R^2^ > 0.99 for all parameters, Fig. 4C). Compared with the reference, the proposed method tends to underestimate longer T1 (> 1400 ms) and slightly overestimates longer T1ρ (> 110 ms). Without B1 correction, sFB-MultiMap caused significant deviation of measured T2 from the reference method and overestimated longer T1ρ. T1 estimations with sFB-MultiMap were also slightly degraded compared with FB-MultiMap. Bland–Altman analyses (Fig. 4D) reveal that the T1 values estimated using the proposed method were a bit lower than the reference (T1 bias = − 71.9 ms, upper 95% limits of agreement = 73.6 ms, lower 95% limits of agreement = − 217.4 ms), whereas T2 and T1ρ values showed better agreement (T2: bias = 1.8 ms, upper 95% limits of agreement = 6.7 ms, lower 95% limits of agreement = − 3.1 ms; T1ρ: bias = 3.9 ms, upper 95% limits of agreement = 13.2 ms, lower 95% limits of agreement = − 5.4 ms).

### In vivo imaging

**Fig. 5:**
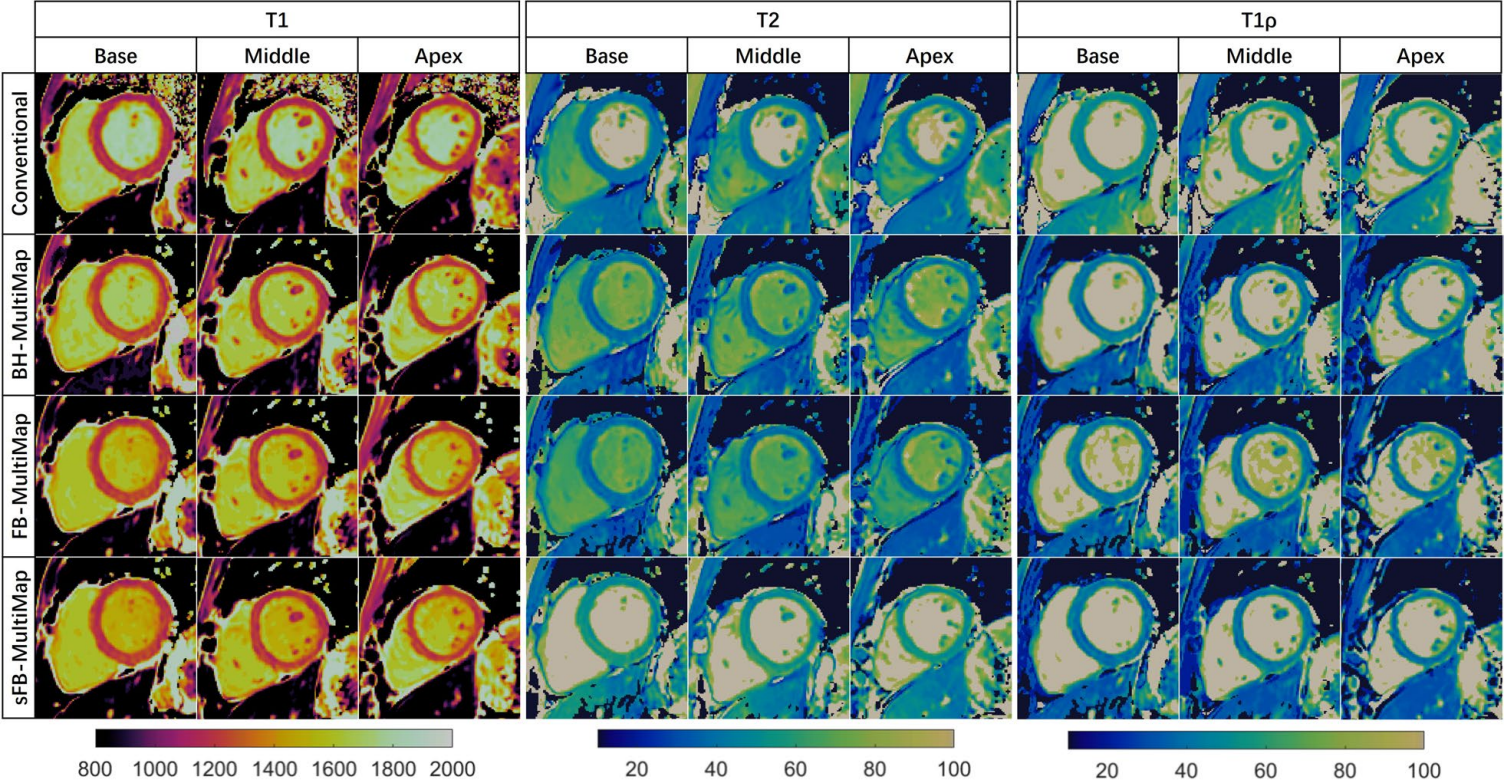
T1, T2 and T1ρ maps at three short-axis slices of a representative healthy subject measured by the conventional breath-hold mapping techniques of MOLLI, T2-prep and T1ρ-prep bSSFP and the proposed multi-parametric mapping technique performed under breath-holding (BH-MultiMap) and free-breathing (FB-MultiMap), and FB-MultiMap with simplified dictionary simulation without modelling B1 (sFB-MultiMap)

**Fig. 6:**
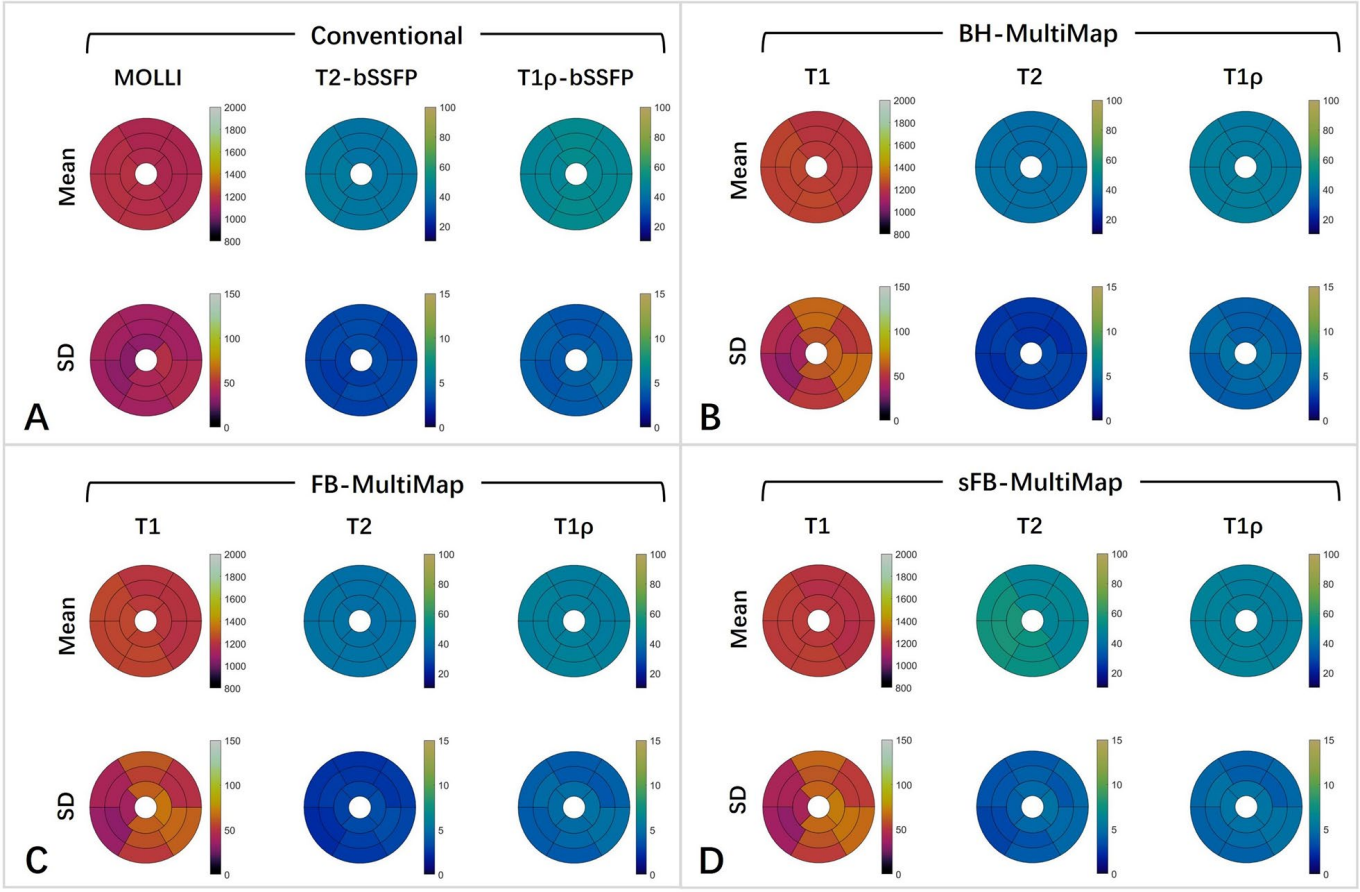
Bullseye plots of different estimation methods including conventional separate breath-hold mapping techniques of MOLLI, T2-prep and T1ρ-prep bSSFP and BH-MultiMap, FB-MultiMap and sFB-MultiMap. The mean and standard deviation (SD) averaged across all healthy subjects for T1, T2 and T1ρ of the 16 segments in the three short-axis slices are shown

**Fig. 7:**
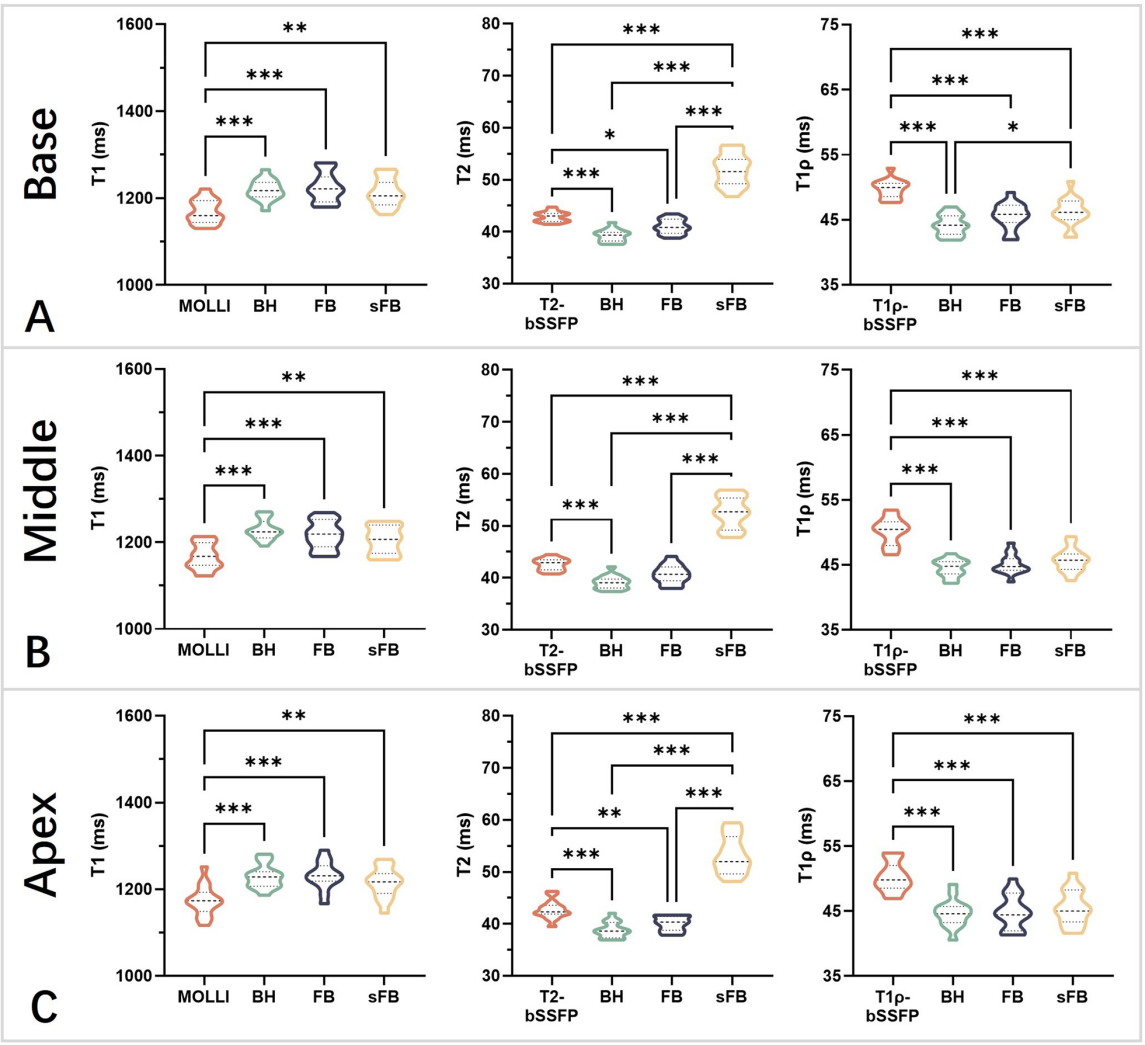
Violin plots of the mean myocardial T1, T2 and T1ρ values for the basal (**A**), middle (**B**) and apical (**C**) shortaxis slices of the 15 healthy subjects estimated with the conventional separate mapping techniques and the multiparametric mapping acquired under breath-holding (BH) and free-breathing with (FB) and without (sFB) B1 correction. The black dashed lines indicate the median values, while the dotted lines represent the first and third quartiles. * indicates statistically significant difference (p < 0.05). ** and *** respectively indicates p < 0.01 and p < 0.001

**Fig. 8:**
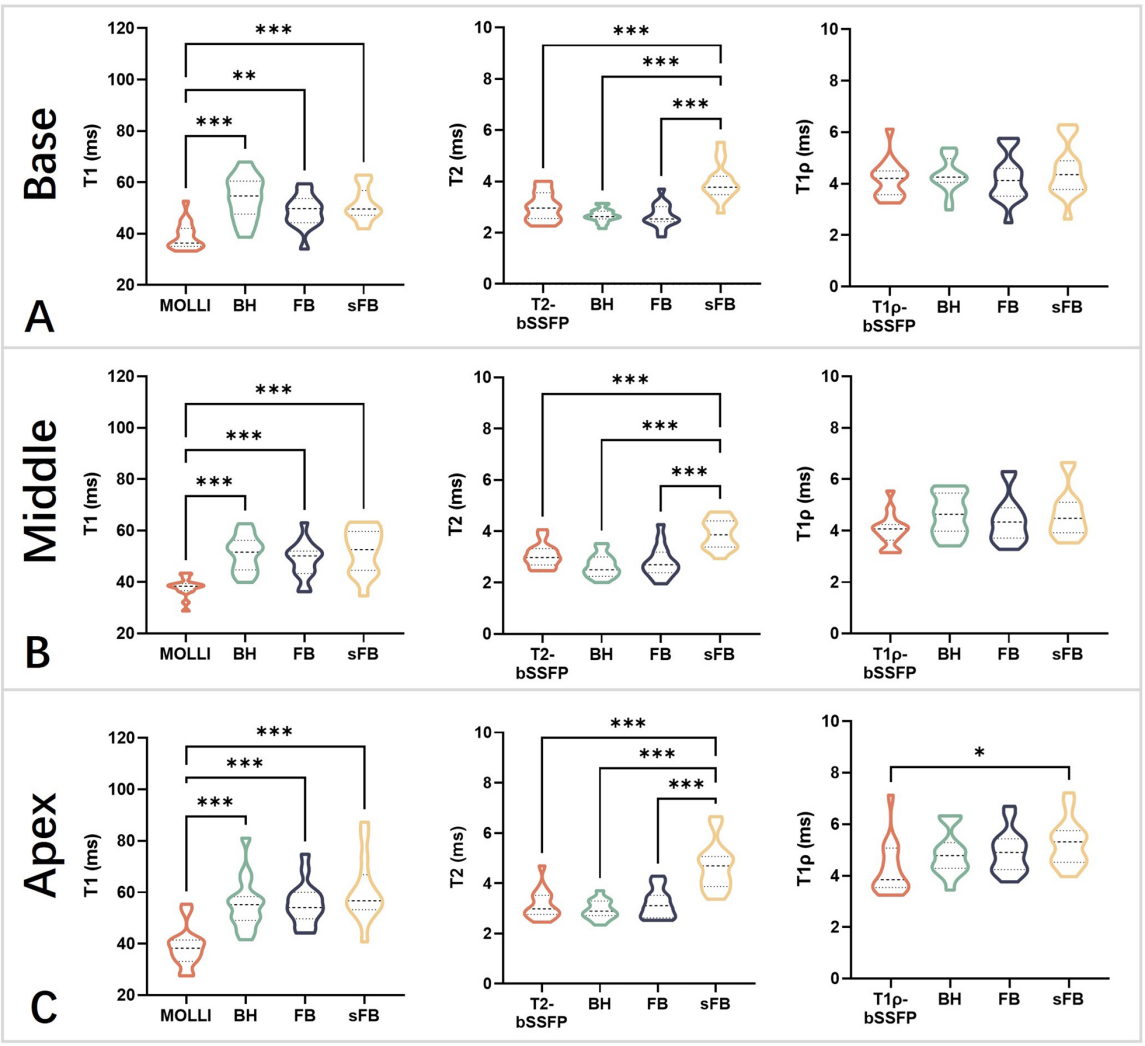
Comparison of the standard deviation of myocardial T1, T2 and T1ρ values for the basal, middle, and apical short-axis slices of the 15 healthy subjects estimated with the four different measurement methods. The black dashed lines indicate the median values, while the dotted lines represent the first and third quartiles. * indicates statistically significant difference (p < 0.05). ** and *** respectively indicates p < 0.01 and p < 0.001

Example T1, T2, and T1ρ maps at three short-axis slices of a representative healthy subject are provided in Fig. 5, where good mapping quality can be observed for FB-MultiMap without discernable motion artifacts compared with conventional mapping methods and BH-MultiMap. Like the phantom results, sFB-MultiMap overestimated T2. The segment-wise analysis results are visualized in Fig. 6 using bullseye plots, where the mean and SD for each segment were calculated by averaging across the 15 healthy subjects. The mapping quality was similar between BH- and FB-MultiMap with overall homogeneous parametric maps while sFB-MultiMap resulted in increased T2 measurement variability compared to FB-MultiMap. The mean and SD of myocardial T1, T2 and T1ρ values of each short-axis slice in the healthy subjects are compared between the four mapping methods in Figs. 7 and 8. There is no significant difference of parameter estimations between BH- and FB-MultiMap for all short-axis slices, while the mean and SD of FB-MultiMap T1 were significantly higher than MOLLI (mean T1 at mid-cavity: 1218 ± 50 ms vs. 1166 ± 38 ms, p < 0.001). The mean T2 and T1ρ estimated with FB-MultiMap was respectively lower than T2-prep bSSFP and T1ρ-prep bSSFP (mean T1ρ at mid-cavity: 45.3 ± 4.4 ms vs. 50.2 ± 4.0, p < 0.001), whereas the difference between FB-MultiMap and T2-prep bSSFP was not significant at the middle short-axis slice (41.2 ± 2.8 ms vs. 42.5 ± 3.1 ms, p = 0.06). The T2 SD of sFB-MultiMap was significantly higher than the other three measurement methods (p < 0.001).

**Fig. 9:**
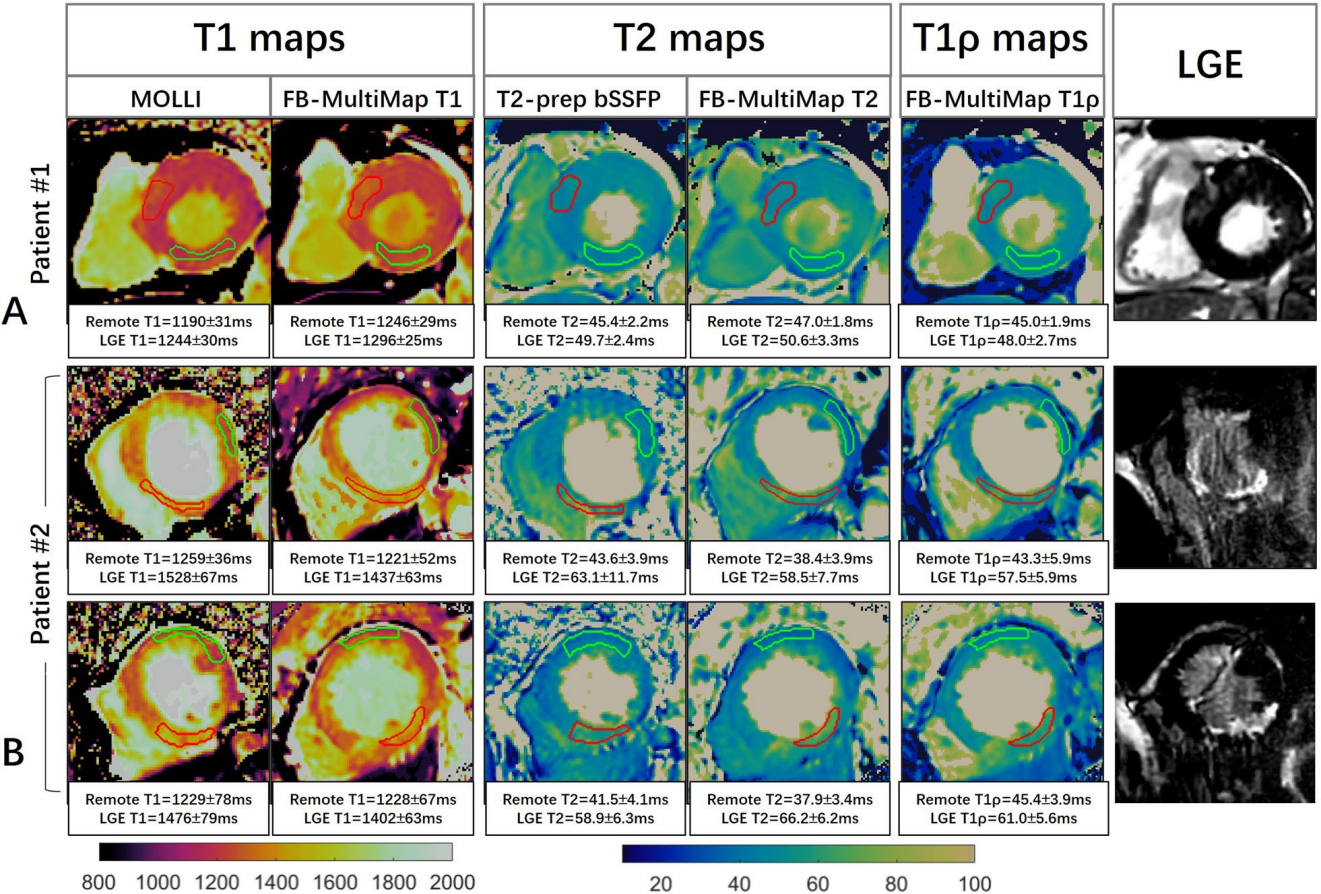
Parameter maps obtained with the proposed FB-MultiMap method and traditional techniques of MOLLI, T2-prep and T1ρ-prep bSSFP, along with the LGE images of two patients. **A** A 79-year-old female patient with hypertensive cardiomyopathy and intramural LGE in the antero-septal segment. **B** A 71-year-old male patient with subendocardial myocardial infarction in the basal and mid-cavity infero-septal segments. The mean and standard deviation of T1, T2 and T1ρ values in the enhancement area (red contour) and remote myocardium (green contour) were reported

Figure 9 shows the parametric maps and slice-matched LGE images of two patients: a 79-year-old female with hypertensive cardiomyopathy and intramural LGE in the antero-septal segment; a 71-year-old male patient with subendocardial myocardial infarction in the basal and mid-cavity inferoseptal segments. For Patient #1, elevated T1, T2 and T1ρ can be observed in the enhancement area (red contour) compared with the remote myocardium (green contour). In this patient, the T1 and T2 values in the remote myocardium were also higher than the healthy subjects, which is not the case for T1ρ, indicating there may be non-fibrotic myocardial abnormality in the remote myocardium. For Patient #2, the T1, T2 and T1ρ values were also increased in the scar region compared with the remote myocardium. This patient was unable to hold his breath well, leading to motion artifacts and motion blurring in the breath-hold LGE images and parameter maps, while the proposed free-breathing multi-parametric mapping technique can still generate good quality parameter maps. The alterations of parameters in the diseased myocardium were consistent between FB-MultiMap and conventional mapping methods.

**Fig. 10:**
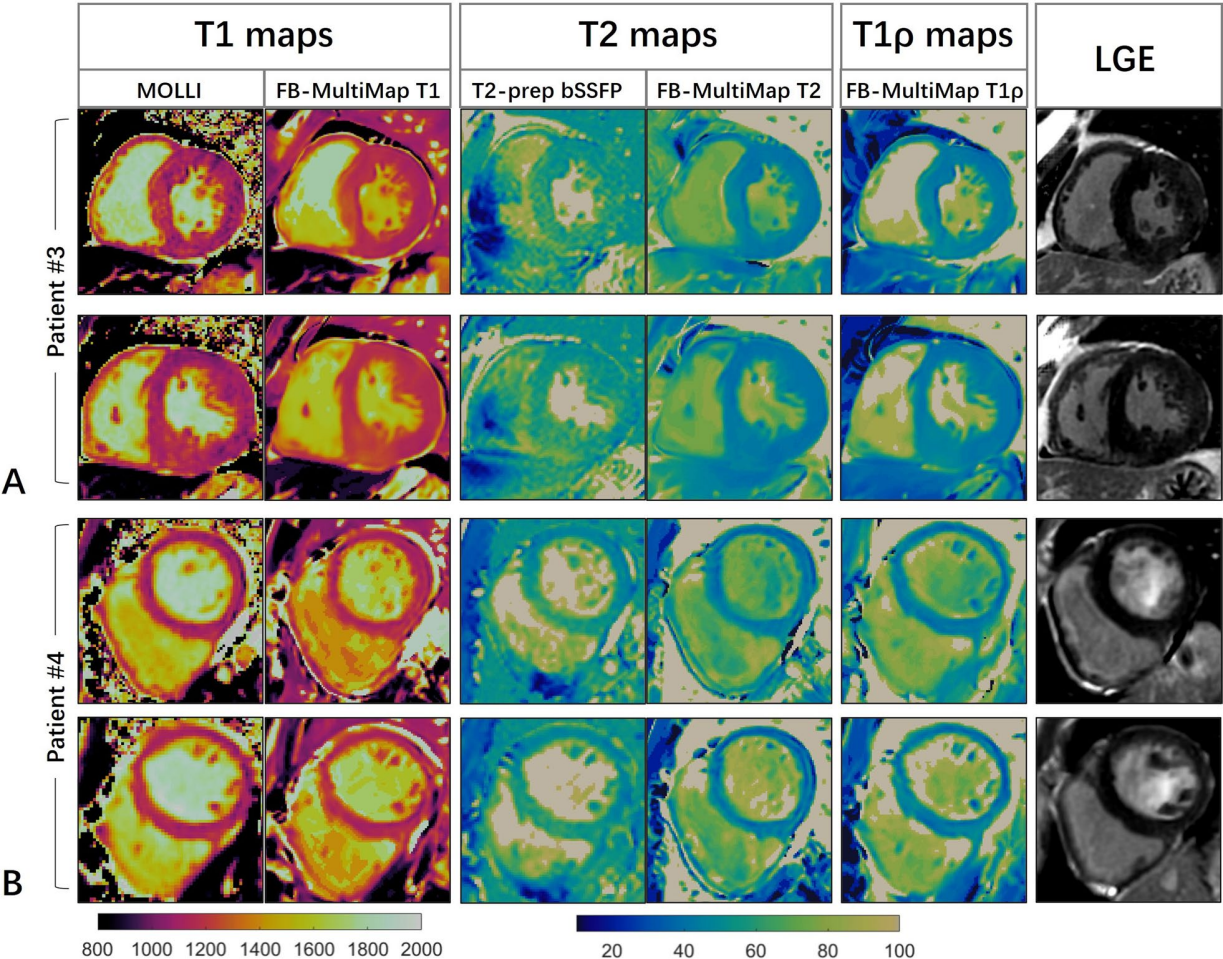
FB-MultiMap and reference parameter maps, and LGE images of two Fabry patients. **A** A 37-year-old male patient with mean myocardial T1 at middle short-axis slice of 1183 ms and 1110 ms, as measured with FB-MultiMap and MOLLI, respectively. **B** A 71-year-old male patient with mean myocardial T1 at middle short-axis slice measured by FB-MultiMap and MOLLI of 1143 ms and 1091 ms, respectively. The mean T1 values of the two patients are shorter compared with the mean T1 of healthy subjects, while T2 and T1ρ values were similar between the patients and healthy subjects in this study

The images of two male patients diagnosed with Fabry disease, aged 37 and 31 years respectively, are demonstrated in Fig. 10. No significant LGE was observed in the two subjects. In Patient #3, except for the basal inferoseptal and middle inferior, the myocardial T1 values were ~ 100 ms lower compared with the average of healthy subjects for both FB-MultiMap and MOLLI. Myocardial T1 values were also significantly shorter as measured with MOLLI and FB-MultiMap in the second Fabry patient (Fig. 10B). The mean T2 and T1ρ of the two Fabry patients were similar to values of healthy subjects in this study. The FB-MultiMap images of the two additional patients with one diagnosed as Fabry cardiomyopathy and another diagnosed as amyloid cardiomyopathy are provided in the Additional file 1.

## Discussion

In this study, a free-breathing multi-parametric mapping technique was proposed for simultaneous myocardial T1, T2, and T1ρ mapping with B1 correction using a single-shot Cartesian acquisition and dictionary matching. The sequence was optimized for the number of preparation pulses, their timing, and variable excitation flip angles in order to obtain accurate T1, T2 and T1ρ estimations in the presence of B1 in homogeneities. In phantoms, the optimized sequence showed good agreement with reference measurements with no discernable dependency on heart rate. In healthy subjects, the free-breathing multi-parametric mapping quality was comparable to standard breath-hold mapping techniques. In preliminary validations in patients, the FB-MultiMap showed promising results for detecting various cardiomyopathies without contrast agent.

The single-shot Cartesian dictionary-based mapping technique has been recently been proposed for simultaneous cardiac T1 and T2 mapping under a breath-hold of ten heartbeats [36]. However, extending this technique to include more relaxation parameters is not straightforward. Firstly, more cardiac cycles are needed for additional parameter sensitization and the scan time may be too long for breath holding. In the proposed technique, a free-breathing acquisition was achieved with dNAV for prospective motion correction and multi-contrast image registration for retrospective motion correction, breaking the limit of breath-hold duration. Secondly, in addition to IR and T2-prep, the T1ρ-prep should be added for T1ρ sensitization, which requires further modification of the configuration of preparation pulses to ensure estimation accuracy and precision. Several candidate sequences with different IR, T2- and T1ρ-prep settings were investigated. The sequence with acquisition of 16 heartbeats (2 IRs, 4 recovery heartbeats between IR and T2/T1ρ-prep, and 4 T2/T1ρ-prep acquisitions) achieved overall lower RRMSE for all parameters in simulations and phantoms and the lowest spatial variability for in vivo parameter maps which indicates better accuracy and precision. Furthermore, the previously proposed Cartesian dictionary-based mapping technique [36] assumed homogeneous B1 across the left ventricle at 1.5T which is not true for 3T. According to our results, the effective B1 was much lower in the septum than in the lateral region. The non-uniformB1 factors, if not considered in dictionary simulations, leaded to inaccurate parameter estimations, especially for T2. The variable flip angle strategy was proposed with flip angles optimized in simulations, phantoms and in vivo imaging, achieving B1 corrected T1, T2 and T1ρ measurements. Similar to MRF, FB-MultiMap generates parameter maps based on dictionary matching. However, the proposed technique involves no complex image reconstruction to remove aliasing artifacts as required in MRF and thus may be more ready adopted in clinical settings for T1, T2 and T1ρ mapping. In addition, most cardiac MRF techniques require breath-holding, including the recent cardiac T1, T2 and T1ρ MRF [39], while the proposed technique allows for free-breathing acquisition. Compared with conventional mapping techniques, such as MOLLI and T2/T1ρ-prep bSSFP, which assumes an analytical model for parameter fitting, the dictionary based multi-parametric mapping method, such as FB-MultiMap, is advantageous in that it requires no empty cardiac cycles for signal recovery, being less dependent on heart rate, and could consider inter-parameter confounders in the signal model, leading to improved quantification accuracy. However, the subject-specific dictionary generation can be time-consuming. To reduce the computation time, simplified Bloch simulations were adopted in the dictionary generation with the B1 factor correcting the excitation flip angle only, without considering imperfect slice profile and preparation pulses. These confounders may be considered for improved accuracy at the expense of significantly increased dictionary simulation times [54, 55].

Similar to MOLLI and T2-prep bSSFP, 2D bSSFP readout was adopted in FB-MultiMap for the benefit signal-to-noise ratio. However, it is noted that bSSFP is sensitive to B0 inhomogeneity which may influence parameter estimation. The influence of off-resonance on the parameter estimations of FB-MultiMap was investigated in the Additional file 1 (Section 4). The off-resonance within ± 100 Hz has little influence on T1, T2 and T1ρ estimation, while B1 is more vulnerable to the B0 inhomogeneity. When the off-resonance exceeds the range of ± 50 Hz, B1 tends to be overestimated. Seeing from the typical in vivo B0 field maps in the Additional file 1: Fig. S7, the B0 offset in the heart region is well below 100 Hz, while in the liver and chest, it could exceed the range of ± 150 Hz. The large off-resonance leads to erroneously high B1 estimations in the non-Cardiac region. Considering the cardiac T1, T2 and T1ρ estimations with FB-MultiMap are robust to off-resonance within ± 100 Hz which can be achieved for most commercial 3T scanners with proper B0 shimming in the heart, the off-resonance was not modelled in the dictionary simulation. The spoiled gradient echo readout is more robust to non-uniform B0, albeit with reduced signal-to-noise ratio. Its feasibility for the multi-parametric mapping technique is worth exploring in future studies.

In phantoms, the proposed FB-MultiMap achieved good accuracy for short to moderate relaxation times. However, it tended to underestimate T1. The underestimation was caused by the uncorrected inversion efficiency in FB-MultiMap. In T1 fitting of IR spin echo, the three-parameter model was used to consider the imperfect inversion. However, in the dictionary simulation of FB-MultiMap, the inversion efficiency was assumed to be 100%, which leaded to the T1 underestimation of FB-MultiMap. FB-MultiMap overestimated longer T1ρ (> 110 ms). Increasing the TSL in T1ρ-prep may help to improve FB-MultiMap estimation accuracy for longer T1ρ.

In healthy subjects, FB-MultiMap achieved similar mapping quality to BH-MultiMap, indicating that after motion correction, respiratory motion has little influence on parameter quantification of FB-MultiMap. The FB-MultiMap T1 was higher than MOLLI, which may suggest improved T1 accuracy of FB-MultiMap as MOLLI is known to underestimate T1 [22, 56]. However, compared with MOLLI, the SD of and BH- and FB-MultiMap T1 was higher. As can be seen in the measurement homogeneity analysis results in the Additional file 1 (Section 5), the septal and inferior T1 was about 30 ms longer compared to the lateral and anterior T1 for both MOLLI and the multi-parametric mapping methods. This difference is likely caused by the non-uniform inversion efficiency. For T1 mapping with MOLLI and FB-MultiMap, the inversion efficiency was assumed to be evenly 100% across the left ventricle myocardium. However, the inversion efficiency can be different due to the non-uniform transmit B1, leading to the slightly different T1 estimations in different myocardial segments. Optimizing the inversion pulse design using for example, the tangent/hyperbolic tangent adiabatic pulse [57] can mitigate the problem of non-uniform inversion efficiency in the presence of inhomogeneous transmitting B1. More details about the influence of inversion efficiency on FB-MultiMap are provided in the Additional file 1 (Section 6). The myocardial T2 and T1ρ values measured by the proposed technique were lower compared to conventional T2 and T1ρ mapping methods that adopt two-parameter fitting, while it is known that the latter methods tend to overestimate T2 and T1ρ due to unaccounted T1 relaxation during readout [58, 59].

Cardiac diseases are usually complex, involving more than one type of pathological change in myocardium. There is where multi-parametric mapping techniques could come into play to aid in diagnosis with comprehensive myocardial tissue characterization. The feasibility of FB-MultiMap in detecting cardiomyopathies has been demonstrated in several patients. The pathological changes detected with FB-MultiMap agreed with conventional breath-hold mapping methods. Specifically, increased T1 and T1ρ values were observed for all the LGE-positive patients in the enhancement area, indicating their potential for detecting myocardial fibrosis without contrast agent. The deposition of lysosomal sphingolipids in Fabry disease leads to low T1 values, which were detected by both FB-MultiMap and MOLLI. In one Fabry patient (Fig. 10A), the basal inferoseptal and middle inferior segments had exceptionally longer T1 (1253 ms) when compared to other segments. There was no LGE in the long T1 regions, while much higher T2 (48.8 ms) and slightly increased T1ρ (47.2 ms) were observed, which may indicate inflammation and early fibrosis. The fibrosis and inflammation may alter the T1 measurement in Fabry disease as reported in previous studies [59, 60]. T1ρ mapping in Fabry disease has not been reported before. FB-MultiMap detected no marke alterations in T1ρ in the three Fabry patients. However, this finding is inconclusive due to the small sample size, and more Fabry patients are needed to validate this point.

### Limitations

There are several limitations in this study. Although efforts were made to optimize the variable flip angle three-parameter mapping technique, the optimization was limited to several empirically designed candidate sequences. Exploring more complex preparation pulse configurations and variable flip angle schemes may help to further improve accuracy and precision. The diaphragm navigator based through-plane motion correction was performed with fixed slice-tracking factor. Adopting a patient-specific motion model trained in a calibration scan to characterize the heart motion relative to dNAV [61] may help to further improve motion correction, especially for apical slices. The proposed technique involves subject-specific dictionary generation which is computationally demanding. Deep learning neural networks may be explored to reduce post-processing time. Finally, this study focused on technical development and only a small number of patients were included for proof-of-concept. Further validation in more patients with cardiovascular diseases is warranted to assess the clinical value of the proposed technique.

## Conclusions

The proposed approach enables simultaneous native myocardial T1, T2 and T1ρ mapping with B1 correction using single-shot Cartesian acquisition and dictionary matching during free breathing. FB-MultiMap demonstrated good agreement with reference techniques in phantoms and achieved similar mapping quality to conventional breath-hold mapping techniques. The multi-parametric mapping technique also demonstrated promising results in detecting pathological changes in T1, T2 and T1ρ. With efficient free-breathing acquisition and simple post-processing, FB-MultiMap has great potential for clinical applications.

### Supplementary Information

**Additional file 1.** Optimization of the multi-parametric mapping sequence, additional results of variable flip angle strategy optimization, additional patients results, influence of off-resonance, and influence of inversion efficiency.

## Data Availability

The datasets used and/or analyzed in the current study will be available from the corresponding author upon reasonable request.

## Abbreviations

AHA: American Heart Association
BH-MultiMap: Breath-hold multi-parametric mapping
bSSFP: Balanced steady-state free precession
dNAV: Diaphragmatic navigator
FA: Flip angle
FB-MultiMap: Free-breathing multi-parametric mapping
FOV: Field-of-view
IR: Inversion recovery
LGE: Late gadolinium enhancement
MOLLI: MOdified Look Locker Inversion recovery
MR: Magnetic resonance
MRF: Magnetic resonance fingerprinting
RRMSE: Relative root mean squared error
RSD: Relative standard deviation
SD: Standard deviation
sFB-MultiMap: FB-MultiMap with simplified post-processing
SL: Spin lock
SSIR: Slice-selective IR
T1ρ-prep: T1ρ preparation
T2-prep: T2 preparation
TSL: Spin lock time
VFA: Variable flip angle
