# Supplementary material for: Free-breathing simultaneous native myocardial T1, T2 and T1ρ mapping with Cartesian acquisition and dictionary matching
Source: J Cardiovasc Magn Reson. 2023 Nov 9;25:63. doi: 10.1186/s12968-023-00973-6 (PMC10636995; doi:10.1186/s12968-023-00973-6)
Supplement: Supplementary file 1 — Additional file 1. Optimization of the multi-parametric mapping sequence, additional results of variable flip angle strategy optimization, additional patients results, influence of off-resonance, and influence of inversion efficiency. [file 12968_2023_973_MOESM1_ESM.docx]

**Additional file 1**

**Section 1: Optimization of the multi-parametric mapping sequence**

Seven candidate sequences with different preparation pulse combinations were empirically designed with 2 or 3 inversions, 2 to 4 recovery cardiac cycles after IR and 3 or 4 T2/T1ρ preparations (Figure S1). For comparison, numerical simulations were performed for typical T1 (1200 ms), T2 (40 ms) and T1ρ (50 ms) of myocardium with heart rate of 80 bpm. Gaussian noise with signal-to-noise ratio of 30, similar to the real acquisitions, was added to simulated signal curves. The simulation for each candidate sequence was repeated 1600 times. Then, all candidate pulse sequences were assessed in phantoms and two healthy subjects. The relative root mean-squared error (RRMSE) for T1, T2 and T1ρ, and the mean RRMSE of the three parameters were computed to evaluate the sequence performance in simulation and phantom studies. The RRMSE is calculated as $\frac{\sqrt{\sum_{i=1}^{N} \left( y_{i}-y \right)^{2}}}{Ny}$, where $y_{i}$ is the i^th^ measurement, $y$ is the ground truth or reference value and N is the total number of measurements. To assess in vivo mapping quality, the relative spatial variability (RSD) was calculated by dividing the standard deviation (SD) with the mean value in the myocardium for T1, T2 and T1ρ.

RRMSE values calculated for the seven candidate sequences are summarized in Table S1. The sequence with two IRs and four recovery cardiac cycles followed by four T2- or T1ρ-preps achieved the lowest mean RRMSE of 0.0343 and the lowest T1ρ RRMSE of 0.0501. For validation in phantoms, RRMSE was calculated for the tubes with T1, T2 and T1ρ values similar to myocardium. The phantom results in Table S2 indicated that except for 2IR-22Re-3Prep, other candidate sequences achieved similarly low RRMSE for T1 and T2, while 2IR-44Re-4Prep had much lower RRMSE for T1ρ and achieved the lowest mean RRMSE. Also, this candidate sequence had overall lower RSD for all parameters in the two tested subjects as shown in Table S3 and Figure S2.


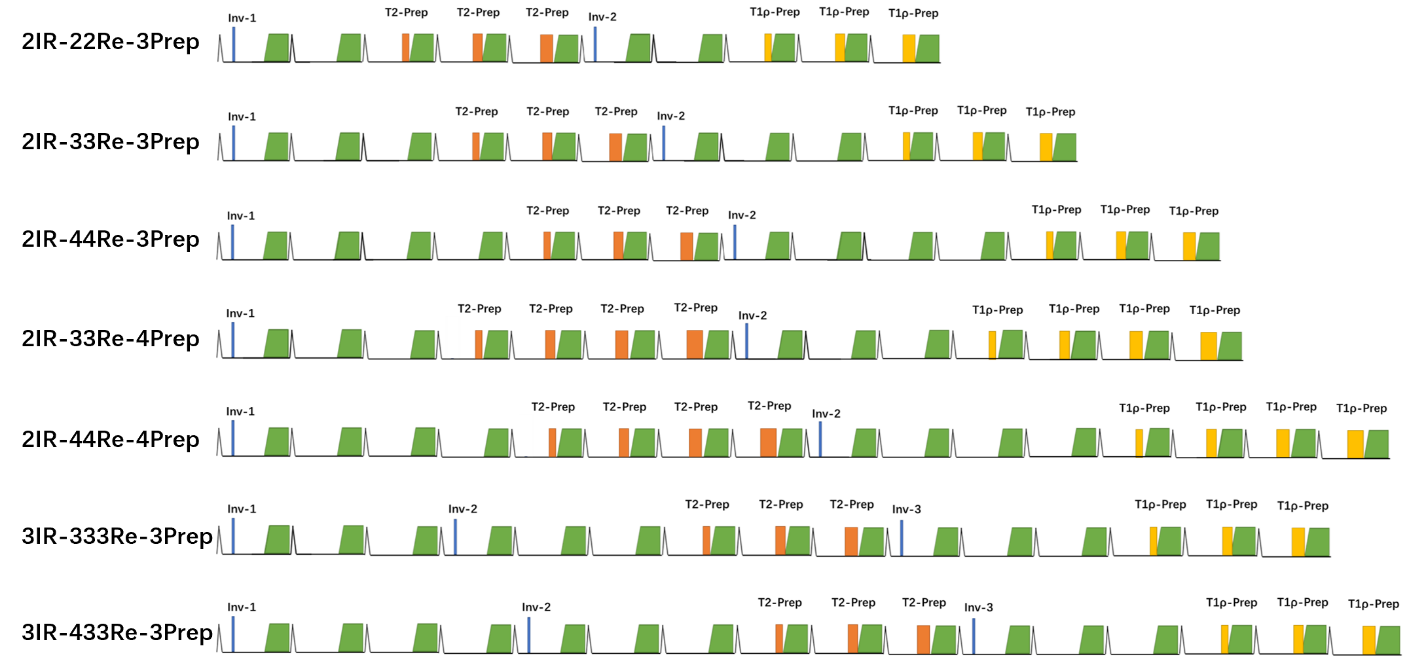


**Figure S1** Diagram of seven candidate sequences with different preparation pulse combinations. The candidate sequence is named according to the total number of inversions (nIR), the number of recovery heart beats after each IR without T2- or T1ρ-prep (nRe) and the number of T2- or T1ρ-preparation pulses after each IR (nPrep). The inversion recovery time is set to 255ms. The durations of T2 preparation for 3Prep and 4Prep are {35, 45, 55} ms and {35, 45, 55, 65} ms, respectively. The spin-lock times of T1ρ preparation for 3Prep and 4Prep are {16, 30, 50} ms and {16, 30, 40, 50} ms, respectively. The longest spin-lock duration is set to 50 ms due to the restrictions of radiofrequency hardware.

**Table S1** The RRMSE of the seven candidate sequences calculated in numerical simulations

| Candidate sequence | T1 RRMSE | T2 RRMSE | T1ρ RRMSE | Mean RRMSE |
| --- | --- | --- | --- | --- |
| 2IR-22Re-3Prep | 0.0172 | 0.0438 | 0.0622 | 0.0411 |
| 2IR-33Re-3Prep | 0.0167 | 0.0394 | 0.0580 | 0.0380 |
| 2IR-44Re-3Prep | 0.0165 | 0.0363 | 0.0552 | 0.0360 |
| 2IR-33Re-4Prep | 0.0168 | 0.0349 | 0.0516 | 0.0344 |
| 2IR-44Re-4Prep | 0.0164 | 0.0363 | **0.0501** | **0.0343** |
| 3IR-433Re-3Prep | 0.0160 | **0.0342** | 0.0570 | 0.0357 |
| 3IR-333Re-3Prep | **0.0158** | 0.0351 | 0.0558 | 0.0356 |

**Table S2** The RRMSE of the seven candidate sequences measured in phantoms

| Candidate sequence | T1 RRMSE | T2 RRMSE | T1ρ RRMSE | Mean RRMSE |
| --- | --- | --- | --- | --- |
| 2IR-22Re-3Prep | 0.0912 | 0.0715 | 0.0822 | 0.0816 |
| 2IR-33Re-3Prep | **0.0349** | **0.0184** | 0.0835 | 0.0456 |
| 2IR-44Re-3Prep | 0.0416 | 0.0241 | 0.0827 | 0.0495 |
| 2IR-33Re-4Prep | **0.0349** | 0.0194 | 0.0859 | 0.0467 |
| 2IR-44Re-4Prep | 0.0351 | 0.0214 | **0.0714** | **0.0426** |
| 3IR-433Re-3Prep | 0.0361 | 0.0254 | 0.0877 | 0.0497 |
| 3IR-333Re-3Prep | **0.0349** | 0.0222 | 0.0860 | 0.0477 |

**Table S3** The mean, SD and RSD of T1, T2 and T1ρ parameters of seven candidate sequences in two healthy subjects

| Subjects | Candidate sequence | T1 Mean±SD (ms) | T2 Mean±SD (ms) | T1ρ Mean±SD (ms) | T1 RSD | T2 RSD | T1ρ RSD | Mean RSD |
| --- | --- | --- | --- | --- | --- | --- | --- | --- |
| Subject #1 | 2IR-22Re-3Prep | 1237±61 | 37.7±3.1 | 44.8±4.0 | 0.0491 | 0.0817 | 0.0890 | 0.0733 |
|  | 2IR-33Re-3Prep | 1219±61 | 37.9±3.1 | 43.1±4.0 | 0.0498 | 0.0811 | 0.0930 | 0.0746 |
|  | 2IR-44Re-3Prep | 1235±59 | 37.3±2.9 | 43.0±3.9 | 0.0476 | 0.0766 | 0.0896 | 0.0713 |
|  | 2IR-33Re-4Prep | 1228±55 | 37.9±2.8 | 43.1±4.3 | 0.0450 | 0.0727 | 0.0875 | 0.0684 |
|  | 2IR-44Re-4Prep | **1226±53** | **37.7±2.4** | **42.5±3.4** | **0.0433** | **0.0642** | **0.0795** | **0.0623** |
|  | 3IR-433Re-3Prep | 1220±62 | 38.1±2.5 | 43.6±3.7 | 0.0511 | 0.0667 | 0.0839 | 0.0672 |
|  | 3IR-333Re-3Prep | 1227±66 | 38.1±2.8 | 44.6±4.2 | 0.0537 | 0.0748 | 0.0952 | 0.0745 |
| Subject #2 | 2IR-22Re-3Prep | 1296±69 | 37.5±3.4 | 45.6±5.3 | 0.0531 | 0.00903 | 0.1163 | 0.0866 |
|  | 2IR-33Re-3Prep | 1284±70 | 36.8±3.8 | 43.3±4.6 | 0.0550 | 0.1028 | 0.1069 | 0.0882 |
|  | 2IR-44Re-3Prep | 1281±66 | 36.6±3.5 | 42.6±4.2 | 0.0513 | 0.0965 | 0.0999 | 0.0826 |
|  | 2IR-33Re-4Prep | 1271±65 | **36.8±2.8** | 42.6±4.5 | 0.0515 | **0.0773** | 0.1066 | 0.0785 |
|  | 2IR-44Re-4Prep | **1262±55** | 37.8±3.4 | **43.0±3.9** | **0.0439** | 0.0906 | **0.0913** | **0.0753** |
|  | 3IR-433Re-3Prep | 1265±59 | 37.5±3.1 | 44.4±4.8 | 0.0465 | 0.0819 | 0.1078 | 0.0787 |
|  | 3IR-333Re-3Prep | 1260±65 | 38.3±3.4 | 44.6±4.6 | 0.0516 | 0.0900 | 0.0787 | 0.0815 |


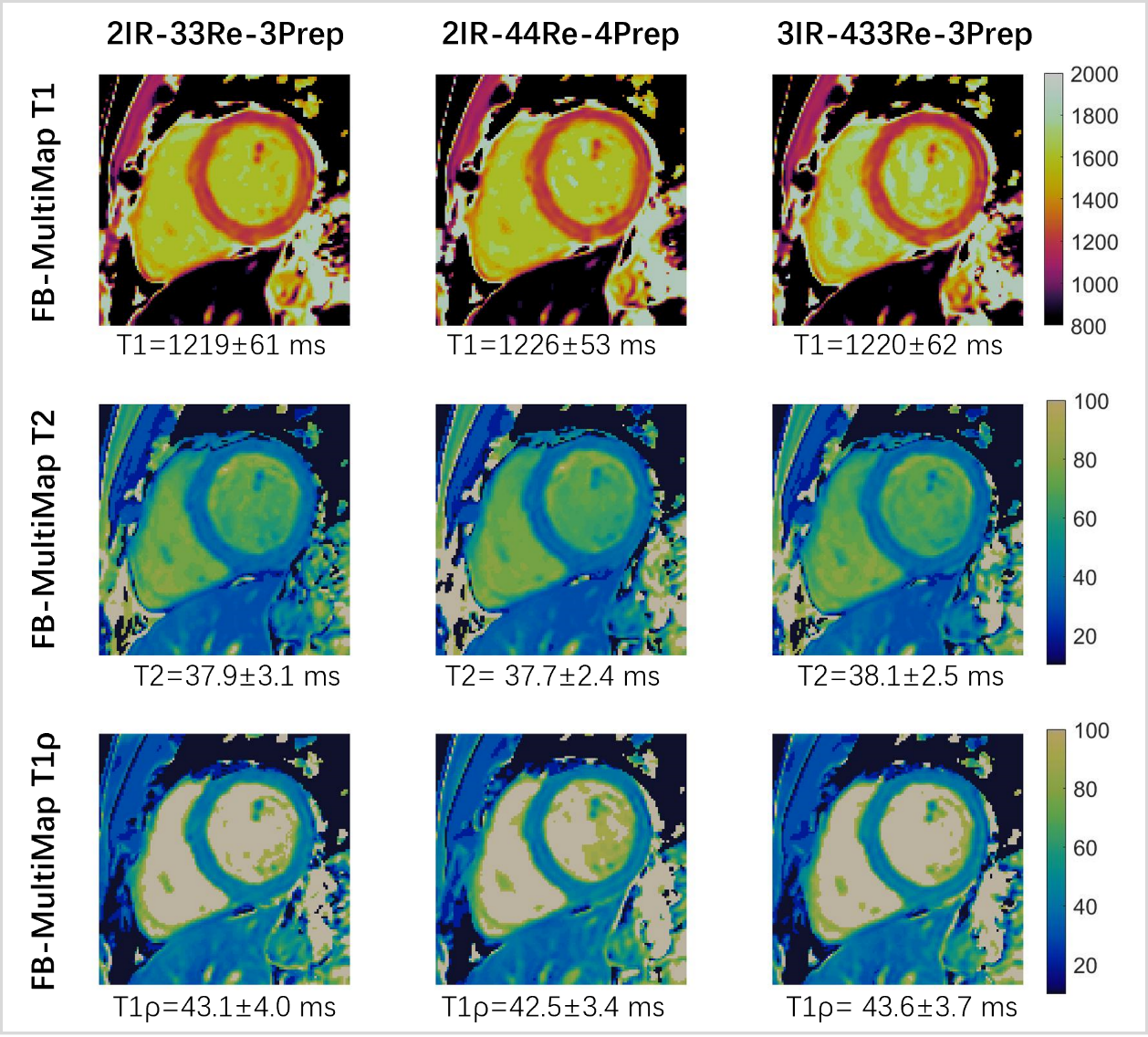


**Figure S2** T1, T2, and T1ρ maps generated using the 2IR-33Re -3Prep, 2IR-44Re-4Prep, and 3IR-433Re-3Prep schemes in one healthy subject. 2IR-44Re-4Prep achieved the lowest spatial variability in the myocardium for all parameters.

**Section 2: Additional results of variable flip angle strategy optimization**

Table S4 summarizes the RRMSE for the constant flip angle scheme, the dual flip angle schemes that lead to the lowest RRMSE for each parameter and the derived four flip angle scheme. The four flip angle strategy achieved the lowest T1 RRMSE and mean RRMSE. Further validation in phantoms (Table S5) also supports the finding of numerical simulations.

**Table S4** The RRMSE of different flip angle combinations in numerical simulations

| Candidate flip angle combinations | | | | T1 RRMSE | T2 RRMSE | T1ρ RRMSE | B1 RRMSE | Mean RRMSE |
| --- | --- | --- | --- | --- | --- | --- | --- | --- |
| FA1-1 (°) | FA1-2 (°) | FA2-1 (°) | FA2-2 (°) |  |  |  |  |  |
| 35 | 35 | 35 | 35 | 0.0195 | 0.0665 | 0.0657 | 0.0822 | 0.0585 |
| 45 | 45 | 70 | 70 | **0.0168** | 0.0550 | 0.0684 | 0.0336 | 0.0435 |
| 35 | 35 | 50 | 50 | 0.0180 | **0.0533** | **0.0574** | 0.0423 | 0.0428 |
| 35 | 35 | 70 | 70 | 0.0180 | 0.0554 | 0.0710 | **0.0312** | 0.0439 |
| 45 | 35 | 70 | 50 | **0.0168** | 0.0568 | 0.0598 | 0.0319 | **0.0413** |

**Table S5** The RRMSE of different flip angle combinations in phantoms

| Candidate flip angle combinations | | | | T1 RRMSE | T2 RRMSE | T1ρ RRMSE | Mean RRMSE |
| --- | --- | --- | --- | --- | --- | --- | --- |
| FA1-1 (°) | FA1-2 (°) | FA2-1 (°) | FA2-2 (°) |  |  |  |  |
| 35 | 35 | 35 | 35 | 0.0473 | **0.0313** | 0.0815 | 0.0534 |
| 45 | 45 | 70 | 70 | 0.0478 | 0.0483 | 0.0532 | 0.0498 |
| 35 | 35 | 50 | 50 | 0.0460 | 0.0395 | 0.0756 | 0.0537 |
| 35 | 35 | 70 | 70 | **0.0423** | 0.0362 | 0.0703 | 0.0496 |
| 45 | 35 | 70 | 50 | 0.0470 | 0.0412 | **0.0469** | **0.0450** |

**Section 3: Additional patient results**

**
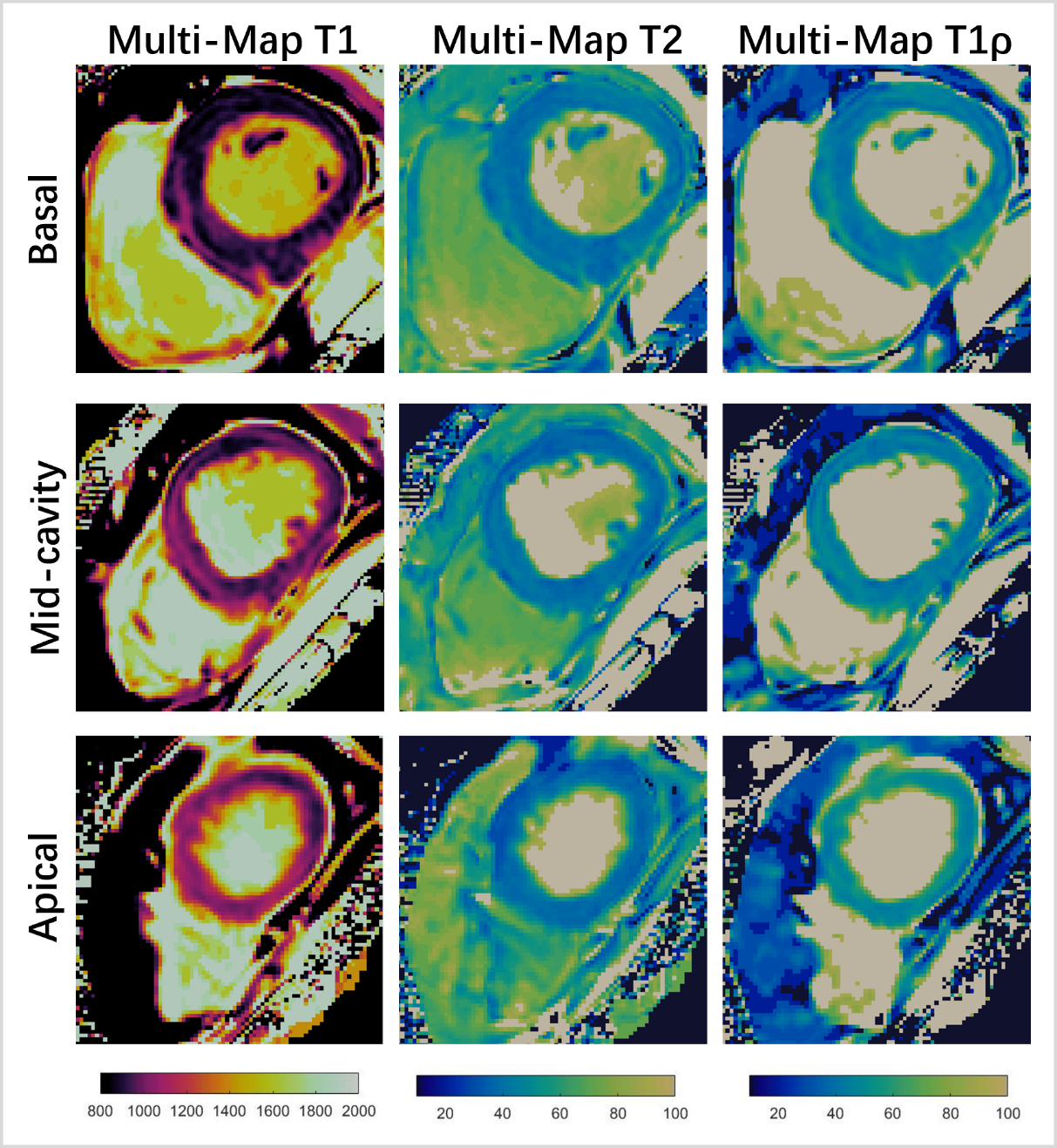
**

**Figure S3** FB-MultiMap parameter maps at three short-axis slice locations in a 72-year-old male patient with Fabry disease (Patient #5). Significantly lower T1 was observed compared with healthy subjects (mean mid-cavity T1: 973 ms vs. 1223 ms).


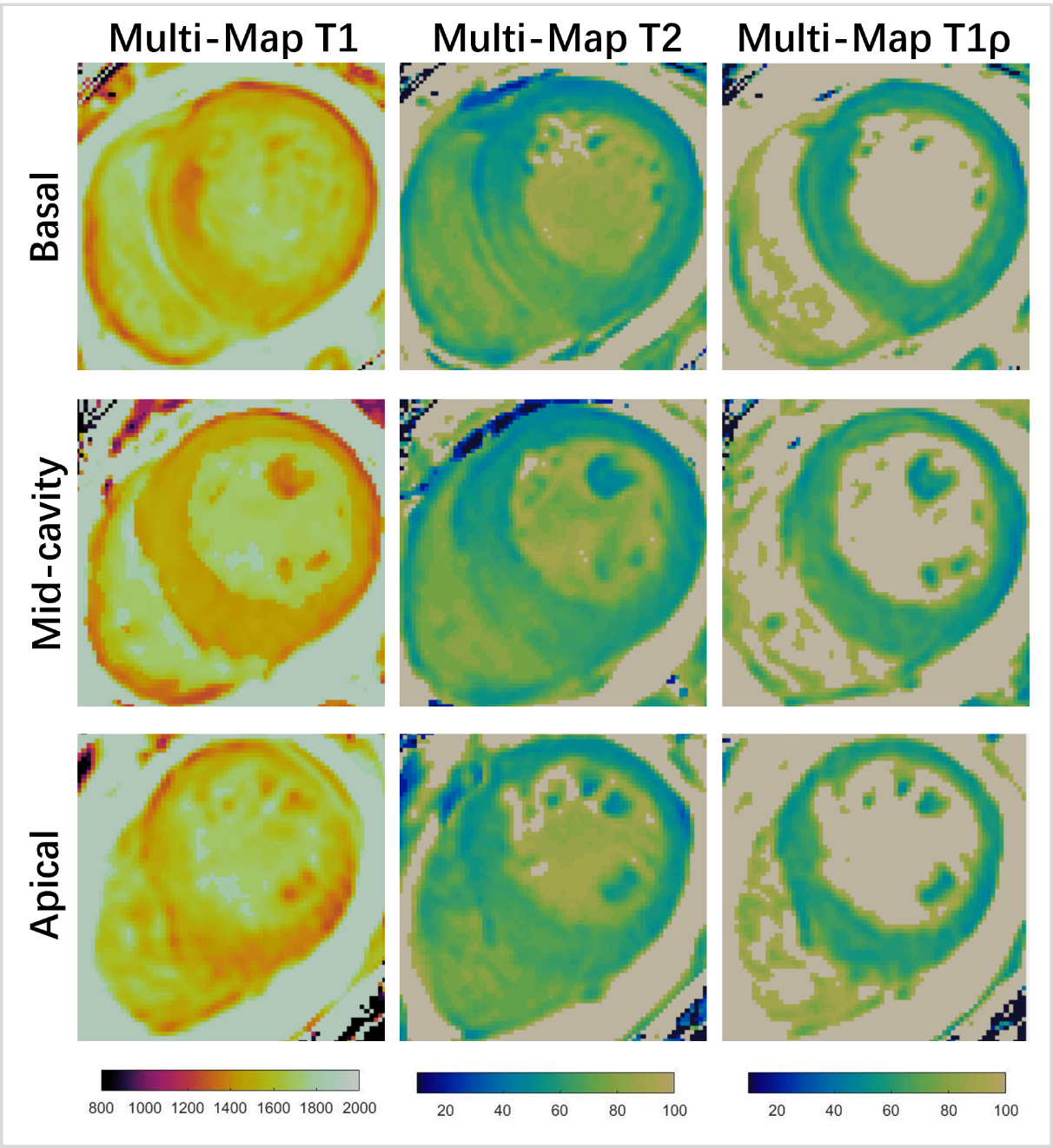


**Figure S4** FB-MultiMap parameter maps at three short-axis slice locations in a 57-year-old male patient with cardiac amyloidosis (Patient #6). Significantly higher T1 (1455 ms), T2 (60.5 ms) and T1ρ (62.5 ms) values were observed for this patient compared with those of healthy subjects (T1, 1223 ms; T2, 40.8 ms; T1ρ, 45.4 ms) at the middle short-axis slice.

**Section 4: Influence of off-resonance**

To investigate the influence of off-resonance on the parameter estimations of FB-MultiMap, it was performed with manually set off-resonance from 0Hz to ±150Hz with a step of ±25Hz and ±200Hz. As the results shown in the following Figure S5 and S6, the off-resonance within ±100Hz has little influence on T1, T2 and T1ρ estimation, while B1 is more vulnerable to B0 inhomogeneity. When the off-resonance exceeds the range of ±50Hz, B1 tends to be overestimated. Typical in vivo B0 maps as well as parameter maps are provided in Figure S7 for two healthy subjects. The B0 offset in the heart region is well below 100Hz, while in the liver and chest, it could exceed the range of ±150Hz. The large off-resonance in the non-Cardiac region leads to the erroneously high B1 estimations in consistency with the findings of phantom experiments.


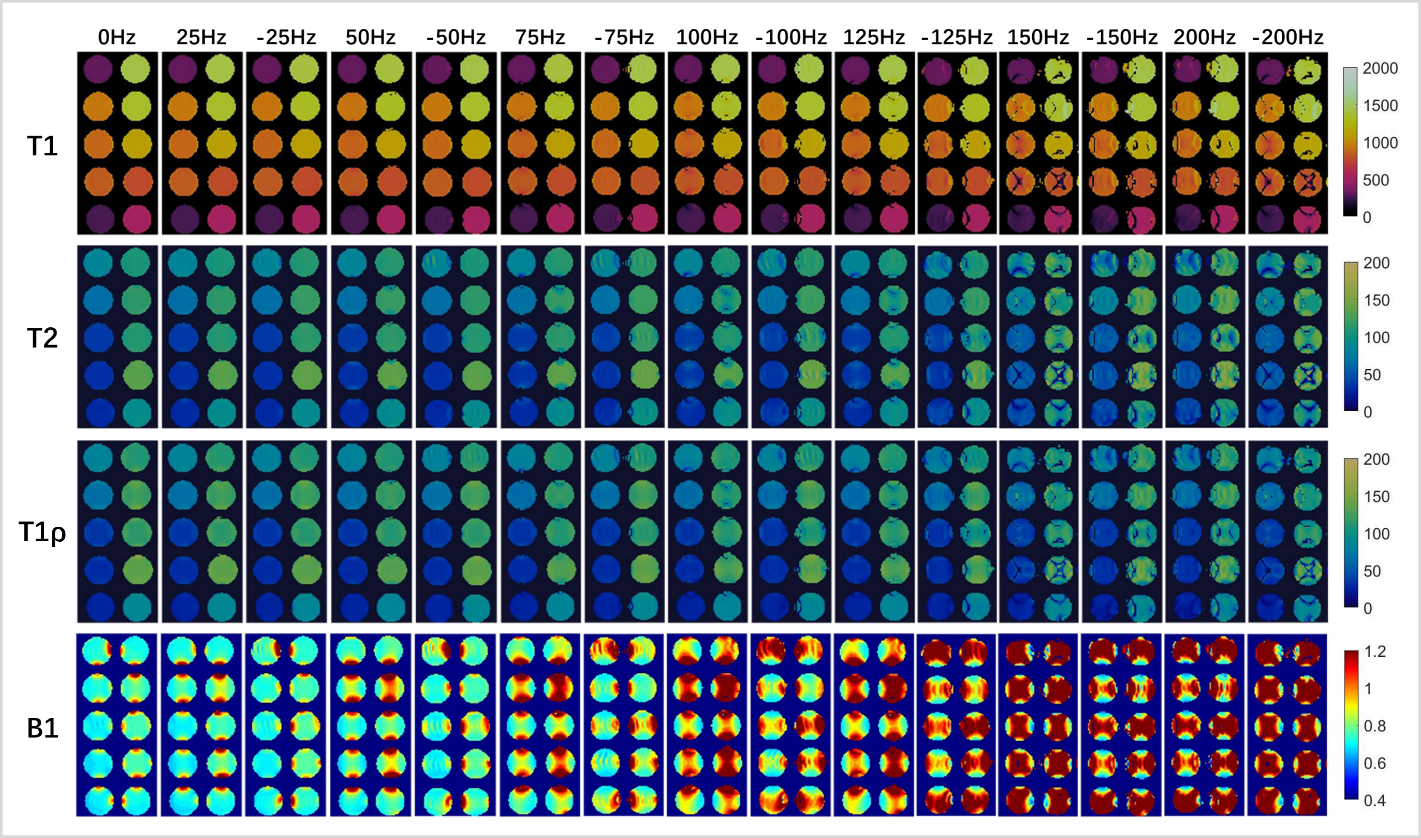


**Figure S5** T1, T2, T1ρ and B1 maps generated by FB-MultiMap at off-resonance from -200Hz to 200Hz. B1 is sensitive to off-resonance and is overestimated in the presence of B0 inhomogeneity.


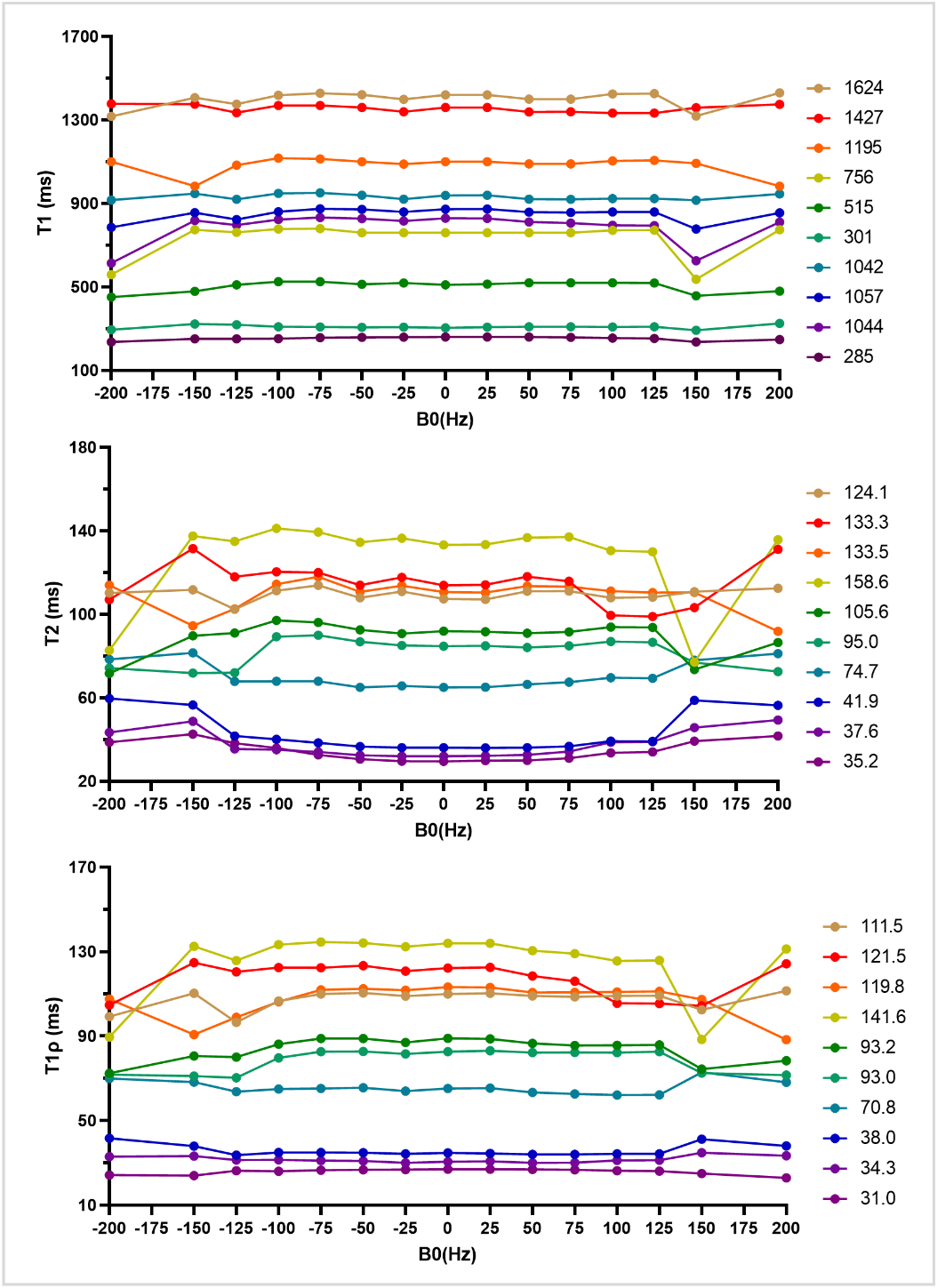


**Figure S6** Phantom T1, T2 and T1ρ values estimated with FB-MultiMap at off-resonance from -200Hz to 200Hz.

**
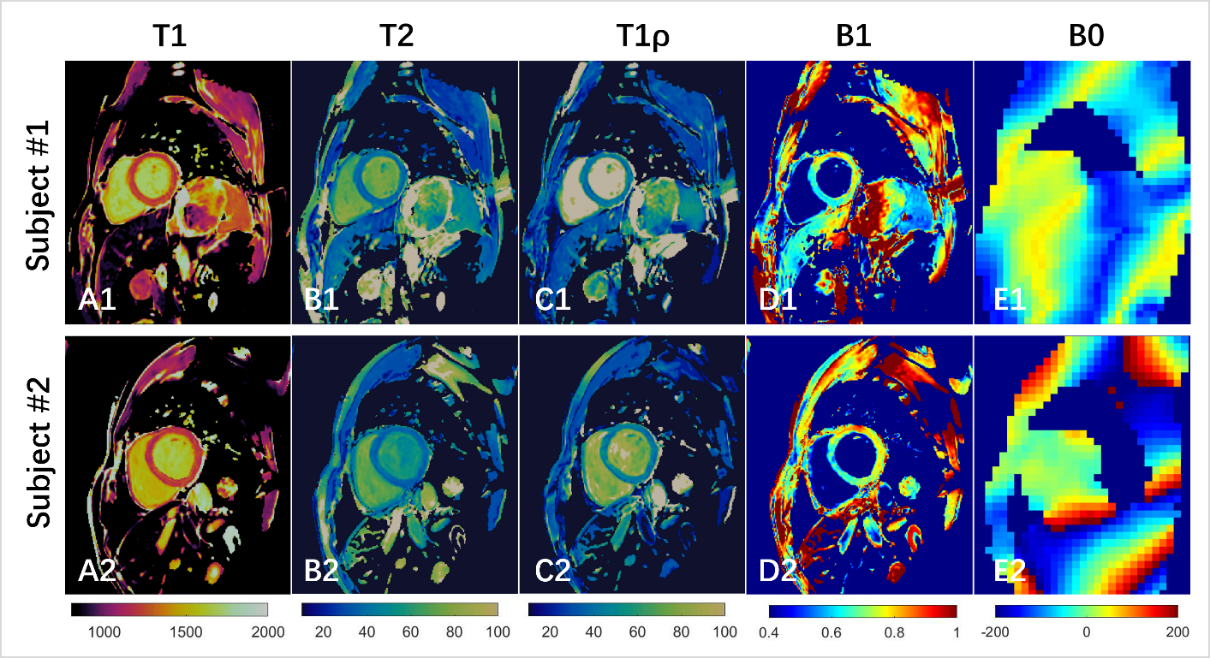
**

**Figure S7** Typical in vivo B0 maps and the estimated T1, T2, T1ρ and B1 maps of two healthy subjects. The off-resonance in the heart region is well below 100Hz which has little influence on FB-MultiMap parameter estimations, while in the non-Cardiac region such as the liver and chest, it could exceed the range of ±150Hz.

**Section 5: Analysis of homogeneity of parameter maps**

The homogeneity of the T1, T2 and T1ρ maps obtained with the conventional separate breath-holding mapping techniques and BH-MultiMap, FB-MultiMap was assessed by comparing the mean parameter values between the three short-axis slices (Base, Middle and Apex) and between the four myocardial segments (Lateral, Septal, Inferior and Anterior) with one-way ANOVA (One-way analysis of variance with Bonferroni post-hoc correction), where the segmental value is calculated by averaging the corresponding segments of the three short-axis slices. As the results summarized in the following Table S6, there were no statistically significant differences between the slices for all parameters of all measurement methods. For segment-wise comparison, the septal and inferior T1 is about 30 ms longer than the lateral and anterior segments for both MOLLI and the multi-parametric mapping methods; the anterior T2 tends to be lower than other segments for all three mapping methods, while the difference is not significant for FB-MultiMap (p=0.08); slightly lower septal T1ρ and lower lateral and anterior T1ρ is respectively observed for T1ρ -bSSFP and the multi-parametric mapping methods.

**Table S6** Comparison of the mean T1, T2 and T1ρ values between the three short-axis slices and between the four segments for the traditional separate mapping techniques, BH- and FB-MultiMap

|  | T1 Mean±SD (ms) | | | T2 Mean±SD (ms) | | | T1ρ Mean±SD (ms) | | |
| --- | --- | --- | --- | --- | --- | --- | --- | --- | --- |
| Slices or Segments | MOLLI | BH-MultiMap | FB-MultiMap | T2-bSSFP | BH-MultiMap | FB-MultiMap | T1ρ-bSSFP | BH-MultiMap | FB-MultiMap |
| Base | 1169±39 | 1218±54 | 1223±50 | 42.8±3.0 | 39.1±2.7 | 41.0±2.7 | 49.8±4.2 | 44.5±4.3 | 45.7±4.2 |
| Middle | 1166±38 | 1229±51 | 1218±50 | 42.5±3.1 | 39.1±2.7 | 41.2±2.8 | 50.2±4.0 | 44.4±4.6 | 45.3±4.4 |
| Apex | 1173±39 | 1228±55 | 1230±58 | 42.6±3.2 | 39.0±2.9 | 40.0±3.2 | 50.4±4.3 | 44.4±4.9 | 45.5±4.9 |
| p value ^a^ | 0.85 | 0.41 | 0.69 | 0.85 | 0.64 | 0.17 | 0.77 | 0.87 | 0.93 |
| Lateral | 1152±42 | 1210±60 | 1200±58 | 42.1±3.1 | 39.1±2.8 | 40.8±3.1 | 50.1±4.4 | 43.6±4.7 | 44.6±4.6 |
| Septal | 1186±35 | 1242±39 | 1246±36 | 43.8±3.0 | 39.6±2.7 | 41.4±2.6 | 48.9±4.0 | 44.9±4.7 | 45.8±4.4 |
| Inferior | 1185±41 | 1240±54 | 1243±55 | 43.0±3.0 | 39.3±2.9 | 41.0±3.0 | 51.2±4.2 | 45.5±4.5 | 46.2±4.4 |
| Anterior | 1153±36 | 1206±64 | 1206±57 | 41.3±3.3 | 37.8±2.6 | 39.6±2.8 | 51.1±3.8 | 43.7±4.3 | 44.6±4.3 |
| p value ^b^ | 0.001* | <0.001* | <0.001* | <0.001* | 0.01* | 0.08 | 0.02* | 0.008* | 0.08 |

^a^ one-way ANOVA of mean values of the three short-axis slices

^b^ one-way ANOVA of mean values of the four segments

* indicates statistically significant difference (p < 0.05)

**Section 6: Influence of inversion efficiency**

In dictionary simulation of FB-MultiMap, the inversion efficiency was assumed to be evenly 100% across the LV myocardium. However, the inversion efficiency can be inhomogeneous due to the non-uniform transmit B1. The inversion pulse in FB-MultiMap is the same to the vendor provided MOLLI to make the two techniques comparable, which is an adiabatic hyperbolic secant (HS) pulse with the following pulse parameters: B1 amplitude=19.86uT, bandwidth=1872Hz, μ=9, duration=20 ms. According to the measured B1 map, the septal transmit B1 is lower than the lateral region. Setting typical B1 of 0.7 and 0.9 respectively for the septal and lateral region, the corresponding inversion efficiency calculated with Bloch simulation (considering T1 and T2 relaxation during the IR pulse with myocardial T1=1300 ms and T2=40 ms) is 0.90 and 0.88. To see how this will influence T1 estimation, two signal curves were simulated for T1=1300 ms, T2=40 ms and T1ρ=50 ms with inversion efficiency of 0.90 and 0.88 and heart rate=80 bpm. Then, the signals were matched to the dictionary generated with perfect inversion efficiency, resulting in 1168/39.6/50.1 ms (T1/T2/T1ρ) for inversion efficiency of 0.90, and 1142/39.4/49.8 ms for inversion efficiency of 0.88. It can be seen the uncorrected heterogeneous inversion efficiency resulted in the slightly different T1 estimations in the septal and lateral myocardium.

The currently estimated B1 in FB-MultiMap is a compound factor, reflecting not only the transmit B1 field, but also other factors such as slice profile effect of the 2D excitation pulse and potential respiratory motion-induced deviations between the measured and simulated signal evolutions. The compound B1 factor cannot be directly used to calculate the inversion efficiency in dictionary simulations. Optimizing the inversion pulse design can mitigate the problem of non-uniform inversion efficiency. For example, with the tangent/hyperbolic tangent pulse of parameters: B1 amplitude=15.21uT, sweep frequency=9500Hz, ξ=10, tan(κ)=22 and duration=5.12ms, the calculated inversion efficiency is 0.93 for both B1 factors of 0.7 and 0.9, resulting in homogeneous T1 estimations in the myocardium.
